# Bodily ownership and agency sensations in a natural state

**DOI:** 10.1038/s41598-021-87843-2

**Published:** 2021-04-21

**Authors:** Souta Hidaka, Kyoshiro Sasaki, Toshikazu Kawagoe, Nobuko Asai, Wataru Teramoto

**Affiliations:** 1 Department of Psychology, Rikkyo University, 1-2-26, Kitano, Niiza-shi, Saitama, 352-8558 Japan; 2 Faculty of Informatics, Kansai University, 2-1-1, Ryozenji-cho, Takatsuki, Osaka 569-1095 Japan; 3 Department of Social Relations, Kyoto-Bunkyo University, 80 Senzoku, Makishima-cho, Uji, Kyoto 611-0041 Japan; 4 Department of Psychology, Kumamoto University, 2-40-1 Kurokami, Chuo-ku, Kumamoto, 860-8555 Japan

**Keywords:** Psychology, Human behaviour

## Abstract

Our bodily sensation is a fundamental cue for our self-consciousness. Whereas experimental studies have uncovered characteristics of bodily sensation, these studies investigated bodily sensations through manipulating bodily sensations to be apart from one’s own body and to be assigned to external, body-like objects. In order to capture our bodily sensation as it is, this questionnaire survey study explored the characteristics of bodily sensation using a large population-based sample (*N* = 580, comprising 20s to 70s age groups) without experimental manipulations. We focused on the sensations of ownership, the feeling of having a body part as one’s own, and agency, the feeling of controlling a body part by oneself, in multiple body parts (the eyes, ears, hands, legs, nose, and mouth). The ownership and agency sensations were positively related to each other in each body part. Interestingly, the agency sensation of the hands and legs had a positive relationship with the ownership sensations of the other body parts. We also found the 60s age group had a unique internal configuration, assessed by the similarity of rating scores, of the body parts for each bodily sensation. Our findings revealed the existence of unique characteristics for bodily sensations in a natural state.

## Introduction

Our body is one of the fundamental cues for our self-consciousness. The body is “always there”^1^ so that “everywhere in the world, self starts with body”^2^. The sensation of having a body, “embodiment,” has been demonstrated as consisting of multiple types of information like sensory signals from vision, audition, vestibular sensation, and interoception, as well as somatosensory sensations and internal representations and models regarding our body^3–5^.

Embodiment is proposed to have at least three sub-components: sensations of self-location, ownership, and agency^6,7^. Specifically, the sensations of ownership and agency are regarded as key components and vigorously investigated in experimental situations^8^. The sensation of ownership represents the feeling that a body part is one’s own (e.g., “This is my hand”), and that of agency reflects the feeling of controlling a body part by oneself (e.g., “I am moving my hand”). The existence and characteristics of these sensations have been demonstrated with bodily illusions. One representative example is the “rubber hand illusion (RHI)”: When we receive spatiotemporally corresponding visual and tactile inputs on a rubber hand and our own hand (out of one’s sight), respectively, these multimodal inputs make us feel as though the rubber hand is our own hand^9^. People feel the sensations of ownership and agency toward the rubber hand when the illusion is introduced^6,10^. Similar bodily illusions have also been reported for a foot^11^ and face^12^. Visuo-tactile synchronous stimulations to a single body part was also reported to induce the illusory ownership sensation of whole body to a body-like object^13–18^.

Since our body itself and bodily sensations are always there, experimental studies have introduced discrepancies in bodily sensations between our own body and external objects such as a rubber hand in order to examine the characteristics of bodily sensations using objective (e.g., proprioceptive drifts) and subjective (questionnaires) measurements^3,4,7,8^. These attempts have successfully uncovered perceptual characteristics of bodily sensations. However, the experimental approaches have some limitations. First, experimental studies have solely focused on instantly altered bodily sensations, namely, how bodily sensations are susceptible to temporally provided manipulations in experimental setups. These approaches have contributed to understanding the existence and malleability of bodily sensations. However, our bodily sensations are not transient, but rather stable in daily life. Thus, the findings obtained from experimental studies may not directly apply to understanding how our bodily sensations are maintained in a natural, ecologically valid manner. Additionally, most experimental studies so far has also focused on a single body part or on the whole body^but see^^15,18^. This may be because the sensations of a single body part, such as the hands, are easy to temporally manipulate, in addition to the fact that experimental manipulations usually take several minutes to induce bodily illusions. Our body, of course, consists of multiple parts and these body parts coordinate with each other. Furthermore, we can feel the sensations of ownership and agency for multiple body parts. This includes body parts that usually move (e.g., hands, eyes, mouth, legs) and those without explicit movements like the ears and nose, but with active, control sensations, such as "listening" and "sniffing" behaviors, respectively.

To the best of our knowledge, no studies have investigated bodily sensations without any experimental manipulations, as well as the relationships of bodily sensations with multiple body parts. Such investigations are necessary to understand how our body is represented in the brain “as it is.” It has been demonstrated that the sensations of bodily ownership and agency can be assessed using a questionnaire during the RHI^6^, although, as mentioned above, the experimental studies solely focused on manipulated bodily sensations. We considered that a survey study could assess bodily sensations in a natural state. A survey study without experimental manipulations further enables us to assess bodily sensations in a large population. Most previous experimental studies use a limited sample size (~ 50 participants, although there are some exceptions^e.g.,^^6,19–21^). This may be due to the fact that experimental manipulations of bodily illusions require relatively longer time. Findings obtained from a survey study with a large data set can contribute to providing novel insights and hypotheses regarding the research on bodily sensations.

The primary aim of this study was to exploratory investigate the bodily ownership and agency sensations in a natural state for multiple body parts. We conducted an online survey with a large dataset (*N* = 580). We used the questionnaire battery developed for measuring bodily sensations under the RHI^6^ with minimum modifications. The target body parts were eyes, ears, nose, mouth, hands, and legs. Experimental and neuroimaging studies have demonstrated that the sensations of bodily ownership and agency are independent in terms of perceptual and neural characteristics, while they also mutually and closely interact with each other^8,10,22^. RHI studies have demonstrated that the illusory sensation of ownership can be induced by both active hand movements and passive somatosensory stimulations. In contrast, the illusory sensation of agency is reported to be triggered solely by active hand movements in the RHI^22–24^. These aspects simply imply that the sensation of ownership is ubiquitous even without body movements, but the agency sensation occurs additionally and occasionally when our body is moving. On the other hand, it has also been demonstrated that feeling ownership can contribute to inducing the agency sensation in RHI^25–28^ and that the sense of agency is reported to enhance the sense of ownership in RHI^22,29^ and to induce a faster motor response to a visually displayed hand with the ownership sensation^23^. These findings indicate that the interactions of these bodily sensations are not unidirectional, but rather mutual. We thus investigated the relationships between the sensations of bodily ownership and agency bidirectionally using a linear regression model: we set the ownership and agency sensations as explanatory variables and each agency and ownership as a target variable, respectively. We also examined “internal configurations” among the multiple body parts in each bodily sensation based on the relative proximity among each body part as a qualitative aspect of the bodily sensations.

The relationships between bodily sensations and demographic variables such as age and gender were also of interest to the current study. Somatosensory^30^ and multisensory processing^31^ have been reported to change (usually worsen) with aging. Further, self-evaluations of attractiveness and appearance are reported to differ across age groups as well as gender^32,33^. We could assume that bodily sensations may also differ with age. However, experimental findings regarding the effects of age on the senses of ownership and agency have been inconsistent: some studies have reported that senior people are more^34^ or less susceptible^19,21,35^ to the body transfer illusion, such as the RHI. In contrast, other studies have demonstrated no age effects^20,36,37^. These differences may be due to large individual differences regarding the susceptibility to body transfer illusions as well as the incongruency of the definitions of age categories and types of illusions. The effects of gender have also been inconsistent among the studies of body transfer illusions^6,29,38^. The current study explored the effects of ages and gender on bodily sensations. We employed the survey method as these demographic variables were almost equally distributed in our data set.

In daily life, we sometimes feel discomfort such as pain, numbness, or tremors in our body parts. Artificially introduced immobilization of a hand was reported to increase the susceptibility to RHI^39^, suggesting that feeling discomfort would degrade the ownership sensation of one’s own body parts. We explored whether and how discomfort sensations were related to the senses of ownership and agency in a natural body state.

## Methods

### Participants

We designed our data collection such that the data of every 100 participants were assigned to either a 20s, 30s, 40s, 50s, 60s, or 70s age group, and gender was equally distributed across the age groups (600 participants’ data in total). We collected the data of 776 participants using the database and platform of an online survey company (Cross Marketing, Japan), but excluded those of 176 participants from analyses since their strategy was regarded as inappropriate (“Satisfice”) by a filler question (see “Questionnaires” section for details). We also found that 20 participants marked the same value for all questionnaire items, even the reversed items. We thus further excluded these data. As a consequence, each age group consisted of 98 (49 females, one participant provided a “not applicable” response for gender), 97 (50 females), 94 (48 females), 96 (50 females), 98 (50 females), and 97 (49 females) participants for 20s, 30s, 40s, 50s, 60s, and 70s, respectively. The age of the participants ranged from 21 to 78 years (mean: 49.38 years). The survey was conducted from June 25 to 29, 2020, in Japan. All procedures were approved by the Ethics Committee of the Department of Psychology, Rikkyo University (Reference number: 20–28), and were performed in accordance with the approved guidelines and the Declaration of Helsinki. Informed consent was obtained from each participant before participation.

### Questionnaires

We gave the instructions to the participants as follows (excerpt from the section related to the study questionnaires): ” The questionnaire regarding body image investigates how we feel about our own body. We will ask you questions about your own body and daily-life experiences. We also will ask you about your age, gender, educational background, and so on. We will ask about your impressions regarding each part of your body, such as your eyes and ears, and about your daily life activities.”

As demographic information, we asked our participants to report their age and gender. The years of education were reported to have a negative relationship with cognitive functions^40^. In Japan, the years of education tended to be shorter for older people. We asked our participants to report the years of education as a control variable for age from one of the following alternatives: 9 (junior high school graduate), 10, 11, 12 (junior high school graduate), 13, 14 (junior college graduate), 15, and over 16 years (university graduate). We also asked them to report if they were using any orthotic equipment such as eyeglasses.

Subjective magnitudes of the sensations of bodily ownership and agency were assessed using the items developed for measuring bodily sensations during the RHI^6^ with minimum modifications (Supplementary information S1). Our questionnaires comprised three ownership items (“I feel like it [a target body part] is my own”, “I feel like it is not mine” [reversed item], and “I feel like it is somebody else’s [reversed item]) and two agency items (“I feel like I am in control of it” and “I feel like it is out of my control” [reversed item]). Each item was scored on a 7-point Likert scale from 1 (strongly disagree) to 7 (strongly agree) with a middle point 4 (Neither agree nor disagree). The target body parts were a left eye, right eye, left ear, right ear, left hand, right hand, left leg, right leg, nose, and mouth. We asked our participants to consider that “hand” indicated the region from the shoulder to the fingertips instead of the part just from the wrist to the fingertips simply because the movements of the hand, wrist, elbow, and arm are usually cooperative. For the same reason, we defined that “leg” indicated the region from the hip to the toes instead of the part just from the ankle to the toes. We also included daily-use goods (shoes and clothes) as a comparison against the body parts. We chose these objects because they were commonly used in daily life and ownership and agency sensations were assumed to occur for them. We further asked our participants to rate “how much discomfort (such as pain, numbness, or tremors) do you feel” for each target body part on a 7-point scale. For the goods targets, we included the item “please check the right-most answer in this question” as the filler question to detect the use of an inappropriate (“Satisfice”) strategy. The presentation order of the targets was randomized for each participant. The order of the questionnaire items was also randomized but consistent among all targets for each participant.

We assumed that activity level in daily life would affect bodily sensations, especially for elderly people. We asked the participants to evaluate “whether you can do it [target activity] by yourself if you try” regarding 13 daily activities using a simple dichotomous rating (yes/no) with an instrumental activity of daily living scale (IADL)^41^: (1) cut toenails, (2) go out by oneself, (3) take a bus or train, (4) shop for necessities, (5) transfer money, (6) lookup a telephone number, (7) vacuum, (8) manage money, (9) control medications, (10) manage a house key, (11) cook, (12) use a microwave, and (13) use a gas stove.

### Data analyses

We first converted the reversed item scores. Since the obtained data were not normally distributed, non-parametric statistical methods were mainly adopted. The concordance of the items for each ownership and agency sensation was tested using Kendall's coefficient of concordance. Correlation analyses were performed using Kendall’s rank correlation coefficient. The comparisons of the scores between laterality (left/right) and gender were conducted using the Wilcoxon rank sum test.

The relationships between the sensations of bodily ownership and agency were analyzed using multiple linear regressions. As mentioned in the introduction, we could assume the bidirectional relationships between these sensations. Thus, the analyses were performed by regarding the ownership scores for all body targets as an explanatory variable and the agency score for each body part as target variable, and vice versa. We also included the interaction term between each explanatory variable and age in each regression equation. Centering (subtracting the averages of each variable from each score in that variable) for explanatory variables and age was necessary for controlling the inflation of correlations between each explanatory variable and the interaction term. Consequently, the explanatory variables ranged from negative to positive scores. We thus performed the multiple linear regression analyses with the Gaussian distribution. The mean and SD of the age data were calculated by assuming a uniform distribution. We created the equation model with ± 1 SD of age and performed the multiple linear regression analyses for each model if a significant beta was found for the interaction term.

We also performed multidimensional scaling (MDS) analyses for each bodily sensation both for all participants’ data and each age group data. The MDS is a method for reconstructing the latent spatial structure underlying a set of items given a matrix of pairwise distances between items^42,43^. Since our data was non-metric, we calculated pairwise distances based on non-parametric correlation and estimated internal configurations for the multiple body parts in each bodily ownership and agency sensation.

We also performed the above-mentioned statistical analyses for the discomfort data. Data analyses and visualizations were performed using R software (version 4.0.1)^44^ with ggplot2^45^, reshape2^46^, tidyverse^47^, synchrony^48^, psych^49^, exactRankTests^50^, car^51^, stringer^52^, ggpubr^53^, hrbrthemes^54^, viridis^55^, fitdistrplus^56^, ppcor^57^, and orddom^58^ packages. Alpha level for statistical tests was set at 0.05 with the Bonferroni correction.

## Results

### Ownership and agency scores

**Figure 1:**
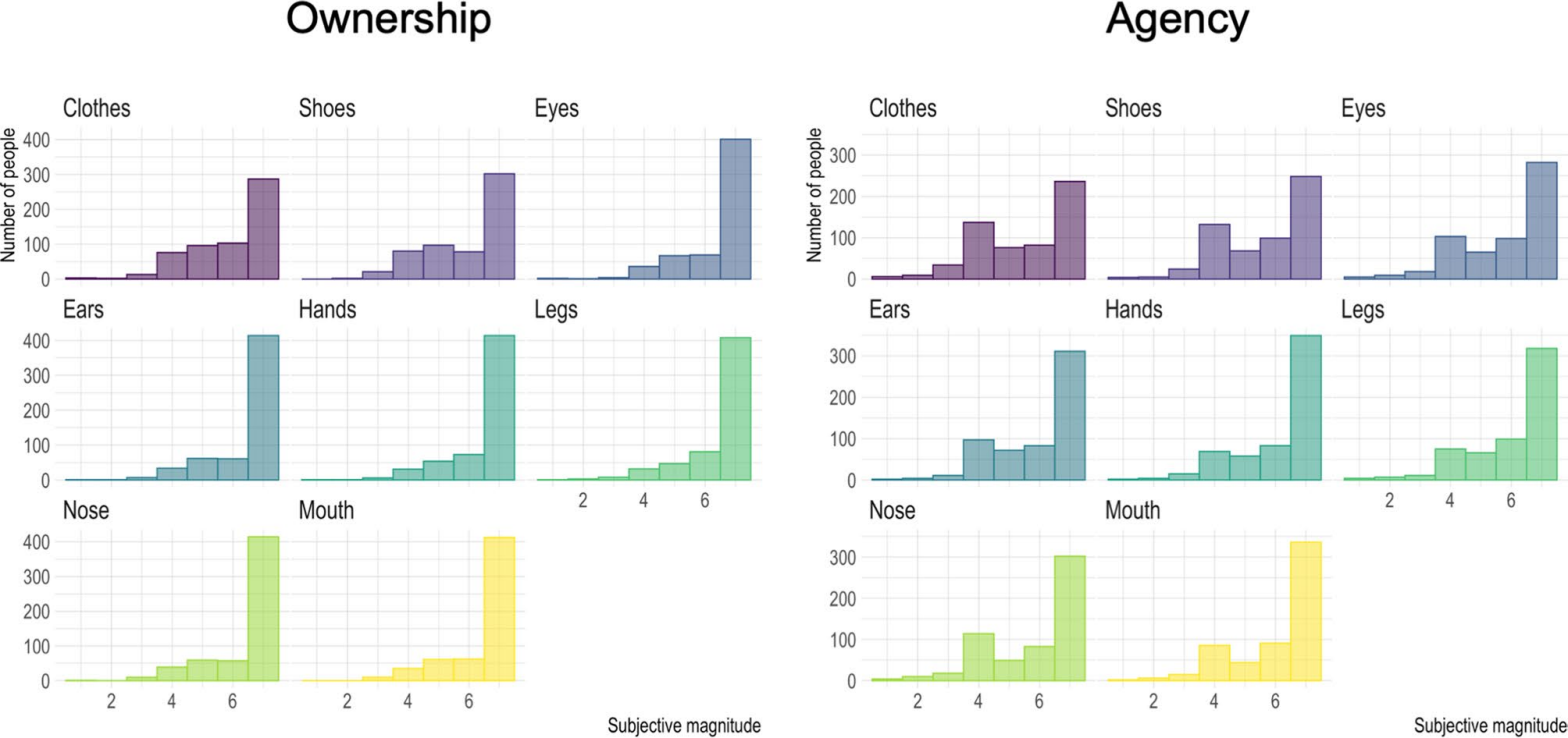
Histograms of the rating score for the ownership and agency sensations in each target. Plots were generated using R software version 4.0.1 (R Core Team (2020). R: A language and environment for statistical computing. R Foundation for Statistical Computing, Vienna, Austria. http://www.R-project.org/).

The rating scores for the questionnaire items showed a significant concordance in each ownership and agency sensation among the body parts and goods targets (*Kendall'*s *W* = 0.61 and 0.58 for ownership and agency, respectively; *ps* < 0.001, one-tailed with 1000 randomized iterations). We averaged the scores for each ownership and agency item for each target. The scores for the targets with laterality (eyes, ears, hands, and legs) were also averaged because there were no statistical differences between the left and right body parts: the Wilcoxon rank sum test (alpha = 0.05/4) found no significant differences for ownership (*ps* > 0.41, *probability of superiority: PSs* < 0.06) and agency (*ps* > 0.06, *PSs* < 0.09) (Supplementary Table S1). The scores of each target were skewed toward “agreed” (Fig. 1). The Shapiro–Wilk test (alpha = 0.05/8) revealed a violation of normality for the ownership (*ps* < 0.001) and agency (*ps* < 0.001) sensations (Supplementary Table S2). This indicates that our participants felt positive bodily sensations regarding the target body parts and objects.

#### Effects of gender

We compared the rating scores for the ownership and agency sensations between males and females for each target. This showed no significant differences (alpha = 0.05/8) both for ownership (*ps* > 0.16, *PSs* < 0.38) and agency (*ps* > 0.14, *PSs* < 0.38) (Supplementary Table S3).

#### Effects of age and years of education

The correlations (alpha = 0.05/8) were not significant between participants’ years of education and the rating scores for the ownership (*Kendall’s τs* < |0.08|, *ps* > 0.07) or agency (*Kendall’s τs* < |0.03|, *ps* > 0.35) sensations (Supplementary Table S4).

We found no significant correlations (alpha = 0.05/8) between participants’ age and the scores for the ownership or agency sensations of the body parts (*Kendall’*s *τs* < 0.07, ps > 0.007) (Supplementary Table S5). In contrast, there were significant positive correlations between age and the goods targets in each ownership and agency score (*Kendall’s τs* > 0.13, *ps* < 0.001). Since there was a significant negative correlation between age and years of education (*Kendall’s τs* < − 0.12, *p* < 0.001), we also performed partial correlation analyses between age and each ownership and agency sensation including the years of education as a covariate. We found very similar results as in the simple correlation analyses (Supplementary Table S5), indicating that the effects of the years of education were negligible: the correlations with the ownership scores showed significant positive correlations for the goods (*Kendall’s τs* > 0.11, *ps* < 0.001), but not for the body parts (*Kendall’*s *τs* < 0.07, *ps* > 0.007). Regarding the agency scores, significant positive correlations were observed for the goods (*Kendall’s τs* > 0.13, *ps* < 0.001) but not for the other targets (*Kendall’*s *τs* < 0.08, *ps* > 0.0064).

These results indicate that the elderly participants felt stronger ownership and agency sensations, particularly for the daily-use goods.

#### Relationships between body parts

The correlations between the ownership and agency sensations as well as those within each sensation were all significantly positive across the targets (alpha = 0.05/64 for each correlation matrix, *Kendall’s τs* > 0.33, *ps* < 0.001) (Supplementary Table S6).

We further examined the relationships between the ownership and agency sensations of the body parts using multiple regression analyses (alpha = 0.05/6 for each regression model). We included the interaction terms between participants’ age and each explanatory variable. The analyses of the ownership scores and each agency score revealed significant regressions for all models (*Fs*(12,567) > 24.27, adjusted *R*^2^ > 0.33, *ps* < 0.001) (Table 1; see also Supplementary Figure S1). Multiple regression equations in each model (alpha level = 0.05/12) showed a specific relationship in each body part: Higher ownership sensation of one body part was positively related to the higher agency sensation of that body part when controlling for the effects of the other body parts (Supplementary Figure S2A). We did not find any interaction effect between the ownership scores and age on the agency score.

**Table 1:** Statistics of multiple regression analyses from the ownership sensations to agency sensation in each body part.

Target: agency	Eyes					Ears					Hands					
Model	R2	F-value	p-value			R2	F-value	p-value			R2	F-value	p-value			
	0.33	25.13	0.000			0.37	28.74	0.000			0.46	41.69	0.000			
Explanatory variables: ownership	B	t-value	p-value	95% CI		B	t-value	p-value	95% CI		B	t-value	p-value	95% CI		
Eyes	**0.60**	**7.20**	**0.000**	**0.43**	**0.76**	0.10	1.31	0.191	− 0.05	0.24	− 0.02	− 0.38	0.705	− 0.15	0.10	
Ears	− 0.02	− 0.16	0.873	− 0.20	0.17	**0.73**	**8.43**	**0.000**	**0.56**	**0.90**	0.07	0.91	0.363	− 0.08	0.21	
Hands	0.03	0.31	0.757	− 0.17	0.24	− 0.20	− 2.16	0.031	− 0.39	− 0.02	**0.61**	**7.59**	**0.000**	**0.45**	**0.77**	
Legs	0.15	1.70	0.089	− 0.02	0.32	0.14	1.78	0.075	− 0.01	0.30	0.07	0.98	0.327	− 0.07	0.20	
Nose	0.03	0.47	0.641	− 0.11	0.18	0.17	2.53	0.012	0.04	0.30	0.12	2.10	0.036	0.01	0.24	
Mouth	0.03	0.32	0.751	− 0.14	0.19	− 0.15	− 2.01	0.045	− 0.30	0.00	0.03	0.50	0.617	− 0.10	0.16	
Eyes * age	0.01	1.52	0.130	0.00	0.02	0.01	1.42	0.157	0.00	0.01	0.00	0.50	0.620	− 0.01	0.01	
Ears * age	0.01	1.90	0.058	0.00	0.02	0.00	0.20	0.845	− 0.01	0.01	0.01	2.60	0.009	0.00	0.02	
Hands * age	0.00	0.84	0.399	− 0.01	0.02	0.00	− 0.45	0.654	− 0.01	0.01	− 0.01	− 1.88	0.061	− 0.02	0.00	
Legs * age	− 0.01	− 2.41	0.016	− 0.02	0.00	0.00	0.20	0.840	− 0.01	0.01	0.00	0.61	0.543	− 0.01	0.01	
Nose * age	0.00	− 0.54	0.586	− 0.01	0.01	− 0.01	− 1.87	0.062	− 0.01	0.00	0.00	− 1.47	0.142	-0.01	0.00	
Mouth * age	− 0.01	− 1.19	0.233	− 0.02	0.00	0.00	0.84	0.399	− 0.01	0.01	0.00	− 0.01	0.995	− 0.01	0.01	

Target: agency	Legs					Nose					Mouth					
Model	R2	F-value	p-value			R2	F-value	p-value			R2	F-value	p-value			
	0.48	46.40	0.000			0.33	24.27	0.000			0.35	27.25	0.000			
Explanatory variables: ownership	B	t-value	p-value	95% CI		B	t-value	p-value	95% CI		B	t-value	p-value	95% CI		VIF
Eyes	0.06	0.99	0.324	− 0.06	0.19	0.02	0.23	0.817	− 0.15	0.18	− 0.12	− 1.60	0.111	− 0.27	0.03	3.17
Ears	− 0.06	− 0.82	0.414	− 0.21	0.09	0.01	0.09	0.931	− 0.18	0.20	− 0.05	− 0.55	0.580	− 0.22	0.12	4.17
Hands	− 0.02	− 0.29	0.771	− 0.19	0.14	− 0.12	− 1.15	0.252	− 0.33	0.09	0.13	1.41	0.160	− 0.05	0.31	4.49
Legs	**0.81**	**11.58**	**0.000**	**0.67**	**0.95**	0.18	2.02	0.044	0.00	0.36	0.21	2.63	0.009	0.05	0.36	3.59
Nose	0.11	1.87	0.061	− 0.01	0.23	**0.64**	**8.38**	**0.000**	**0.49**	**0.78**	0.13	1.92	0.055	0.00	0.26	2.75
Mouth	− 0.01	− 0.11	0.913	− 0.14	0.12	0.09	1.02	0.310	− 0.08	0.26	**0.50**	**6.53**	**0.000**	**0.35**	**0.65**	3.22
Eyes * age	0.00	− 0.84	0.401	− 0.01	0.00	− 0.01	− 2.11	0.035	− 0.02	0.00	0.00	− 0.82	0.413	− 0.01	0.01	3.38
Ears * age	0.01	1.75	0.081	0.00	0.02	0.01	1.19	0.236	0.00	0.02	0.01	2.27	0.024	0.00	0.02	3.94
Hands * age	− 0.01	− 1.66	0.097	− 0.02	0.00	0.00	0.05	0.963	− 0.01	0.01	− 0.01	− 1.82	0.069	− 0.02	0.00	4.02
Legs * age	0.00	0.42	0.673	− 0.01	0.01	0.01	2.25	0.025	0.00	0.02	0.01	2.06	0.039	0.00	0.02	3.87
Nose * age	0.00	− 0.19	0.852	− 0.01	0.01	0.00	0.19	0.853	− 0.01	0.01	0.00	− 0.22	0.822	− 0.01	0.01	2.87
Mouth * age	0.01	1.22	0.224	0.00	0.01	− 0.01	− 0.93	0.354	− 0.02	0.01	− 0.01	− 1.28	0.200	− 0.02	0.00	4.03

Bold letters indicate significant beta values.

Multiple regression analyses of the agency scores and each ownership score showed significant regressions for all models (*Fs*(12,567) > 30.53, adjusted *R*^2^ > 0.38, *ps* < 0.001) (Table 2; see also Supplementary Figure S1). In addition to each body part’s specific relationship (Supplementary Figure S2B), we found that the agency scores of the hands and legs had significant effects on all the body parts: the higher agency scores of hands (Supplementary Figure 3A) and legs (Supplementary Figure 3B) were positively related to the higher ownership scores for the other body parts. We did not find any interaction effect between the agency scores and age on the ownership scores.

**Table 2:** Statistics of multiple regression analyses from the agency sensations to ownership sensation in each body part.

Target: ownership	Eyes					Ears					Hands					
Model	R2	F-value	p-value			R2	F-value	p-value			R2	F-value	p-value			
	0.38	30.53	0.000			0.44	38.24	0.000			0.47	43.06	0.000			
Explanatory variables: agency	B	t-value	p-value	95% CI		B	t-value	p-value	95% CI		B	t-value	p-value	95% CI		
Eyes	**0.20**	**5.43**	**0.000**	**0.13**	**0.28**	− 0.05	− 1.41	0.158	− 0.12	0.02	− 0.02	− 0.50	0.618	− 0.08	0.05	
Ears	0.04	0.99	0.322	− 0.04	0.13	**0.29**	**7.29**	**0.000**	**0.21**	**0.37**	0.02	0.44	0.658	− 0.06	0.09	
Hands	**0.14**	**3.16**	**0.002**	**0.05**	**0.22**	**0.26**	**6.23**	**0.000**	**0.18**	**0.34**	**0.41**	**10.54**	**0.000**	**0.33**	**0.48**	
Legs	**0.16**	**3.98**	**0.000**	**0.08**	**0.25**	**0.12**	**2.99**	**0.003**	**0.04**	**0.19**	**0.15**	**4.04**	**0.000**	**0.08**	**0.22**	
Nose	0.03	0.75	0.456	− 0.04	0.10	− 0.04	− 1.28	0.200	− 0.11	0.02	− 0.03	− 0.85	0.398	− 0.09	0.04	
Mouth	− 0.02	− 0.45	0.656	− 0.10	0.06	0.04	1.15	0.252	− 0.03	0.12	0.07	2.08	0.038	0.00	0.14	
Eyes * age	0.00	− 0.23	0.821	− 0.01	0.00	0.00	− 0.37	0.710	− 0.01	0.00	0.00	1.41	0.159	0.00	0.01	
Ears * age	0.00	0.69	0.491	0.00	0.01	0.00	− 0.92	0.359	− 0.01	0.00	0.00	− 1.31	0.192	− 0.01	0.00	
Hands * age	0.00	1.81	0.070	0.00	0.01	0.00	1.67	0.095	0.00	0.01	0.00	− 0.05	0.959	0.00	0.00	
Legs * age	0.00	− 1.71	0.087	− 0.01	0.00	0.00	− 0.95	0.344	− 0.01	0.00	0.00	− 1.67	0.095	− 0.01	0.00	
Nose * age	0.00	− 1.82	0.069	− 0.01	0.00	0.00	1.12	0.261	0.00	0.01	0.00	0.89	0.373	0.00	0.01	
Mouth * age	0.00	0.25	0.801	0.00	0.01	0.00	0.40	0.689	0.00	0.01	0.00	0.76	0.446	0.00	0.01	

Target: ownership	Legs					Nose					Mouth					
Model	R2	F-value	p-value			R2	F-value	p-value			R2	F-value	p-value			
	0.52	52.44	0.000			0.40	33.59	0.000			0.40	32.53	0.000			
Explanatory variables: agency	B	t-value	p-value	95% CI		B	t-value	p-value	95% CI		B	t-value	p-value	95% CI		VIF
Eyes	− 0.02	− 0.76	0.448	− 0.09	0.04	− 0.04	− 0.99	0.323	− 0.11	0.04	0.02	0.63	0.528	− 0.05	0.09	2.43
Ears	0.02	0.42	0.675	− 0.06	0.09	0.04	0.82	0.411	− 0.05	0.12	− 0.04	− 1.04	0.299	− 0.12	0.04	2.69
Hands	**0.14**	**3.66**	**0.000**	**0.07**	**0.22**	**0.20**	**4.61**	**0.000**	**0.12**	**0.29**	**0.18**	**4.30**	**0.000**	**0.10**	**0.27**	2.47
Legs	**0.43**	**11.96**	**0.000**	**0.36**	**0.51**	**0.16**	**3.74**	**0.000**	**0.07**	**0.24**	**0.15**	**3.66**	**0.000**	**0.07**	**0.23**	2.43
Nose	− 0.01	− 0.32	0.749	− 0.07	0.05	**0.23**	**6.31**	**0.000**	**0.16**	**0.30**	0.02	0.60	0.547	− 0.05	0.09	2.31
Mouth	0.08	2.34	0.020	0.01	0.15	0.01	0.36	0.717	− 0.06	0.09	**0.25**	**6.39**	**0.000**	**0.17**	**0.32**	2.33
Eyes * age	− 0.01	− 2.53	0.012	− 0.01	0.00	0.00	− 0.96	0.338	− 0.01	0.00	0.00	− 1.83	0.068	− 0.01	0.00	2.76
Ears * age	0.00	− 0.18	0.859	0.00	0.00	0.00	− 0.16	0.873	− 0.01	0.00	0.00	0.21	0.835	0.00	0.01	2.69
Hands * age	0.00	1.49	0.136	0.00	0.01	0.00	0.39	0.695	0.00	0.01	0.00	1.41	0.160	0.00	0.01	2.63
Legs * age	− 0.01	− 2.63	0.009	− 0.01	0.00	0.00	− 1.94	0.053	− 0.01	0.00	0.00	− 0.26	0.795	− 0.01	0.00	2.54
Nose * age	0.00	0.77	0.439	0.00	0.01	0.00	0.80	0.425	0.00	0.01	0.00	− 0.72	0.473	− 0.01	0.00	2.40
Mouth * age	0.01	2.52	0.012	0.00	0.01	0.00	0.89	0.376	0.00	0.01	0.00	0.87	0.385	0.00	0.01	2.53

Bold letters indicate significant beta values.

#### Internal configurations of body parts in ownership and agency

**Figure 2:**
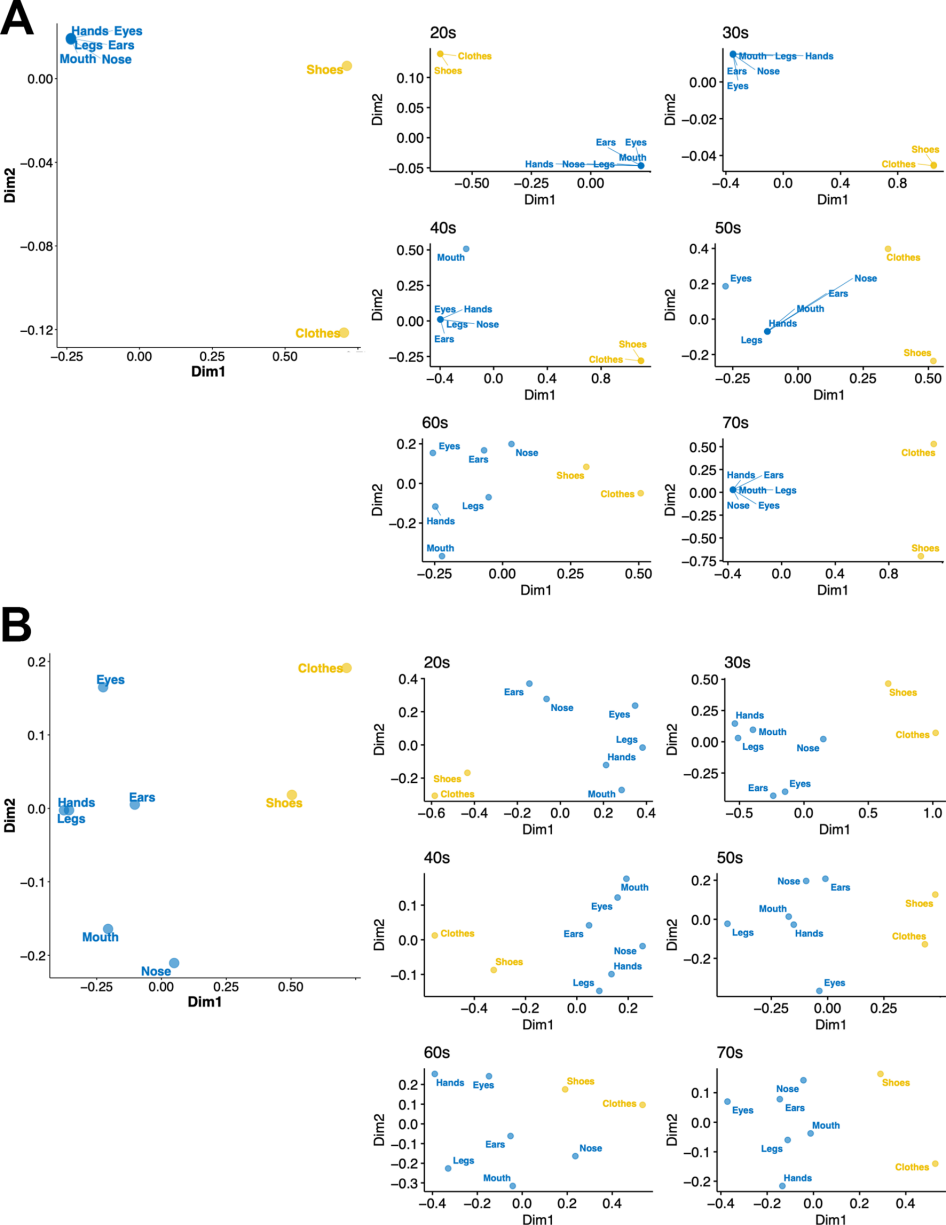
Internal configurations of the targets with multidimensional scaling analyses for (**A**) the ownership and (**B**) agency sensations. The left-side panel shows the whole participants’ data and right-side panels show each age group data. Plots were generated using R software version 4.0.1 (R Core Team (2020). R: A language and environment for statistical computing. R Foundation for Statistical Computing, Vienna, Austria. http://www.R-project.org/).

We performed the MDS analyses to depict 2-D internal configurations of the multiple body parts in each bodily ownership and agency sensation based on the relative distances between each pair of targets. The analyses were applied for all participants’ data and each age group. Regarding the ownership data (Fig. 2A), all body parts were closely located along one arbitrary dimension (Dim 2) and were separated from the object targets along the other arbitrary dimension (Dim 1) in all participants’ data. The very similar tendency was also observed for each age group data except for the 60s age group. In the 60s data, the body parts were also sparse along the one dimension (Dim 2). All participants’ agency sensation data similarly showed that the body part and object targets were clearly separated along one arbitrary dimension (Dim1) (Fig. 2B). This tendency was consistent across the 20s–50s and 70s age group. For the 60s data, however, the eyes, hands, clothes, and shoes were located and grouped along the other dimension (Dim2).

### Discomfort

**Figure 3:**
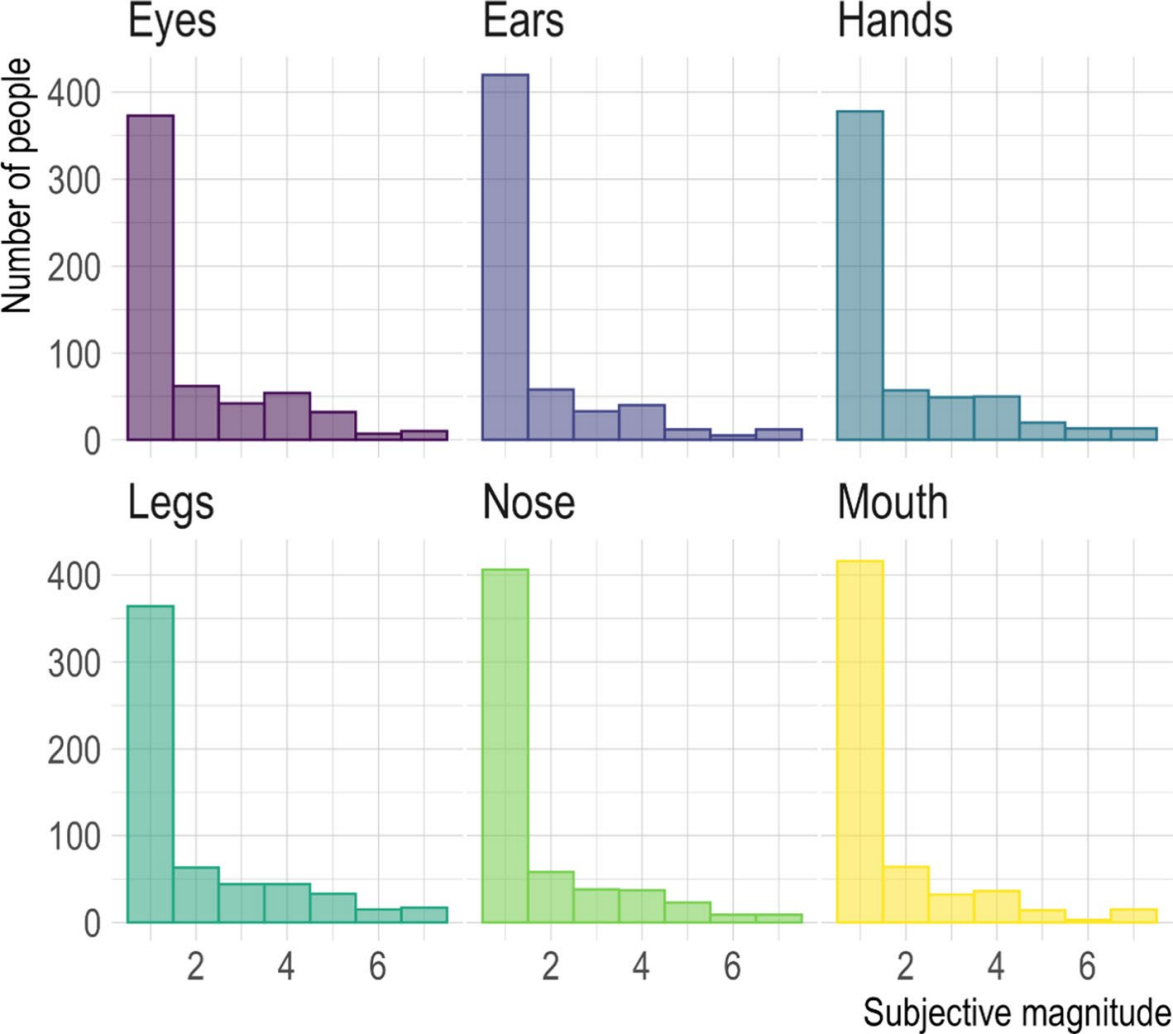
Histograms of the rating score for discomfort in each body part. Plots were generated using R software version 4.0.1 (R Core Team (2020). R: A language and environment for statistical computing. R Foundation for Statistical Computing, Vienna, Austria. http://www.R-project.org/).

We also performed analyses on the discomfort scores. Since the scores were not significantly different (Wilcoxon’s rank sum test, alpha = 0.05/4) between the left and right regarding the body parts with laterality (*Ws* < 1573.5, *ps* > 0.11, *PSs* < 0.07) (Supplementary Table S7), we averaged these scores between the laterality. The scores of each target were skewed toward “disagreed” (Fig. 3). A Shapiro–Wilk test (alpha = 0.05/6) revealed a violation of normality (*Ws* > 0.57, *ps* < 0.001). This indicates that our participants generally felt less discomfort in the body parts (Supplementary Table S8). The correlations of the discomfort scores between the body parts were all significantly positive (alpha = 0.05/15, *Kendall’s τs* > 0.49, *ps* < 0.001) (Supplementary Table S9).

#### Effects of gender, age, and years of education

The comparison of the discomfort scores between males and females (alpha = 0.05/6) showed no significant differences (*ps* > 0.01, *PSs* < 0.35) (Supplementary Table S10). The correlations between each discomfort score and participants’ years of education (alpha = 0.05/6) were not significant (*Kendall’s τs* < |0.06|, *ps* > 0.07) (Supplementary Table S11). No significant correlations were found between each discomfort score and participants’ age (*Kendall’s τs* < |0.06|, *ps* > 0.08) (Supplementary Table S12). There were no significant partial correlations between each discomfort score and age when controlling for the effect of years of education (*Kendall’s τs* < |0.05|, ps > 0.07) (Supplementary Table S12).

#### Relationships among the body parts

We examined the relationships between the discomfort scores and ownership scores or agency scores using multiple regression analyses (alpha = 0.05/6 for each regression model). The discomfort scores for each body part and the interaction term between each discomfort score and age were set as explanatory variables. The analyses for each ownership score revealed significant regressions for all models (*Fs*(12,567) > 13.47, adjusted *R*^2^ > 0.21, *ps* < 0.001) (Table 3; see also Supplementary Figure S4). The multiple regression equations (alpha level = 0.05/12) in each model showed negative relationships in each body part such that stronger discomfort in one body part was associated with lower ownership sensation of that body part when controlling for the effects of the other targets (Supplementary Figure S5A). We also found that the discomfort score for ears was negatively related to the ownership scores for eyes and hands (Table 3; Supplementary Figure S6A). Further, the significant regressions regarding the interaction term between the discomfort scores and age were observed (Table 3; Supplementary Table S13). Regarding the significant interaction term between the discomfort score for nose and age on the ownership score for nose, the multiple regression equations with ± 1SD of age were significant (*F*(3, 576) = 47.28, adjusted *R*^2^ = 0.20, *p* < 0.001). The analyses of the simple slopes with ± 1SD of age (*ps* < 0.001) showed that the effect of the discomfort was stronger (the slope was steeper) for elderly participants (Supplementary Figure S6B). With regard to the significant interaction term between the discomfort score for hands and age on the ownership score for nose, the significant multiple regression equations with ± 1SD of age (*F*(3, 576) = 28.63, adjusted *R*^2^ = 0.13, *p* < 0.001) and the analyses of the simple slopes with ± 1SD of age (*ps* < 0.001) showed that the effect of the discomfort in the hands was stronger (the slope was steeper) for younger participants (Supplementary Figure S6B). Regarding the significant interaction term between the discomfort score for eyes and age on the ownership score for mouth, the multiple regression equations with ± 1SD of age were significant (*F*(3, 576) = 23.54, adjusted *R*^2^ = 0.10, *p* < 0.001). The analyses of the simple slopes with ± 1SD of age (*ps* < 0.001) showed that the effect of the discomfort was stronger (the slope was steeper) for younger participants (Supplementary Figure S6B). For the significant interaction term between the discomfort score for mouth and age on the ownership score for legs, the multiple regression equations with ± 1SD of age were significant (*F*(3, 576) = 41.94, adjusted *R*^2^ = 0.18, *p* < 0.001). However, the estimated simple slopes with ± 1SD of age (*ps* < 0.001) were comparable.

**Table 3:** Statistics of multiple regression analyses from the discomfort to ownership sensation in each body part.

Target: ownership	Eyes					Ears					Hands					
Model	R2	F-value	p-value			R2	F-value	p-value			R2	F-value	p-value			
	0.22	14.93	0.000			0.29	20.23	0.000			0.26	17.54	0.000			
Explanatory variables: discomfort	B	t-value	p-value	95% CI		B	t-value	p-value	95% CI		B	t-value	p-value	95% CI		
Eyes	**− 0.12**	**− 3.49**	**0.001**	**− 0.19**	**− 0.05**	0.02	0.67	0.503	− 0.04	0.09	0.02	0.61	0.542	− 0.04	0.08	
Ears	**− 0.16**	**− 3.98**	**0.000**	**− 0.23**	**− 0.08**	**− 0.26**	**− 7.05**	**0.000**	**− 0.34**	**− 0.19**	**− 0.13**	**− 3.60**	**0.000**	**− 0.20**	**− 0.06**	
Hands	0.03	0.76	0.447	− 0.05	0.10	− 0.02	− 0.62	0.539	− 0.09	0.05	**− 0.11**	**− 3.09**	**0.002**	**− 0.18**	**− 0.04**	
Legs	− 0.08	− 2.46	0.014	− 0.14	− 0.02	− 0.05	− 1.45	0.148	− 0.11	0.02	− 0.06	− 1.82	0.069	− 0.12	0.00	
Nose	− 0.02	− 0.63	0.529	− 0.09	0.05	− 0.03	− 0.90	0.368	− 0.10	0.04	− 0.05	− 1.48	0.139	− 0.12	0.02	
Mouth	− 0.05	− 1.28	0.203	− 0.13	0.03	− 0.10	− 2.71	0.007	− 0.17	− 0.03	− 0.08	− 2.28	0.023	− 0.15	− 0.01	
Eyes * age	0.00	1.76	0.079	0.00	0.01	0.00	2.49	0.013	0.00	0.01	0.00	0.17	0.864	0.00	0.00	
Ears * age	0.00	1.50	0.135	0.00	0.01	0.00	1.98	0.048	0.00	0.01	0.00	1.91	0.056	0.00	0.01	
Hands * age	0.00	− 0.75	0.451	− 0.01	0.00	0.00	0.62	0.539	0.00	0.01	0.00	1.62	0.107	0.00	0.01	
Legs * age	0.00	0.79	0.431	0.00	0.01	0.00	− 0.82	0.415	− 0.01	0.00	0.00	0.68	0.494	0.00	0.00	
Nose * age	0.00	− 1.66	0.097	− 0.01	0.00	− 0.01	− 2.65	0.008	− 0.01	0.00	0.00	− 1.19	0.236	− 0.01	0.00	
Mouth * age	0.00	− 0.68	0.496	− 0.01	0.00	0.00	− 1.28	0.201	− 0.01	0.00	0.00	− 1.88	0.061	− 0.01	0.00	

Target: ownership	Legs					Nose					Mouth					
Model	R2	F-value	p-value			R2	F-value	p-value			R2	F-value	p-value			
	0.31	22.75	0.000			0.25	17.27	0.000			0.21	13.47	0.000			
Explanatory variables: discomfort	B	t-value	p-value	95% CI		B	t-value	p-value	95% CI		B	t-value	p-value	95% CI		VIF
Eyes	0.02	0.48	0.628	− 0.05	0.08	0.03	0.97	0.333	− 0.04	0.10	0.01	0.36	0.718	− 0.06	0.08	2.07
Ears	− 0.09	− 2.42	0.016	− 0.16	− 0.02	− 0.09	− 2.38	0.018	− 0.17	− 0.02	− 0.10	− 2.46	0.014	− 0.17	− 0.02	2.10
Hands	− 0.03	− 0.88	0.382	− 0.10	0.04	0.02	0.42	0.674	− 0.06	0.09	− 0.08	− 1.98	0.048	− 0.15	0.00	2.53
Legs	**− 0.21**	**− 6.82**	**0.000**	**− 0.27**	**− 0.15**	− 0.09	− 2.74	0.006	− 0.16	− 0.03	− 0.06	− 1.79	0.074	− 0.12	0.01	2.13
Nose	− 0.05	− 1.35	0.178	− 0.11	0.02	**− 0.22**	**− 5.96**	**0.000**	**− 0.29**	**− 0.15**	− 0.03	− 0.75	0.455	− 0.10	0.04	1.88
Mouth	− 0.08	− 2.18	0.030	− 0.15	− 0.01	− 0.08	− 2.02	0.044	− 0.16	0.00	**− 0.13**	**− 3.28**	**0.001**	**− 0.20**	**− 0.05**	2.04
Eyes * age	0.00	1.54	0.124	0.00	0.01	0.00	0.12	0.902	0.00	0.00	**0.01**	**2.99**	**0.003**	**0.00**	**0.01**	**1.91**
Ears * age	0.00	2.19	0.029	0.00	0.01	0.00	0.38	0.706	0.00	0.01	0.00	0.01	0.993	0.00	0.00	2.16
Hands * age	0.00	− 0.26	0.796	0.00	0.00	**0.01**	**3.41**	**0.001**	**0.00**	**0.01**	0.00	0.46	0.647	0.00	0.01	2.60
Legs * age	0.01	2.71	0.007	0.00	0.01	0.00	− 0.22	0.823	0.00	0.00	0.00	0.64	0.519	0.00	0.01	2.13
Nose * age	0.00	− 1.83	0.068	− 0.01	0.00	**− 0.01**	**− 3.03**	**0.003**	**− 0.01**	**0.00**	0.00	− 2.25	0.025	− 0.01	0.00	1.81
Mouth * age	**− 0.01**	**− 2.92**	**0.004**	**− 0.01**	**0.00**	0.00	− 1.07	0.284	− 0.01	0.00	0.00	− 1.54	0.124	− 0.01	0.00	2.38

Bold letters indicate significant beta values.

Multiple regression analyses of the discomfort scores and each agency score revealed significant regressions for all models (*Fs*(12, 567) > 9.65, adjusted R^*2*^ > 0.15, *ps* < 0.001) (Table 4, see also Supplementary Figure S4). Multiple regression equations in each model showed negative relationships in each body part (Supplementary Figure S5B). There were no significant interactions between the discomfort scores and age.

**Table 4:** Statistics of multiple regression analyses from the discomfort to agency sensation in each body part.

Target: agency	Eyes					Ears					Hands					
Model	R2	F-value	p-value			R2	F-value	p-value			R2	F-value	p-value			
	0.20	13.33	0.000			0.17	11.15	0.000			0.24	16.23	0.000			
Explanatory variables: discomfort	β	t-value	p-value	95% CI		β	t-value	p-value	95% CI		β	t-value	p-value	95% CI		
Eyes	**− 0.33**	**− 6.69**	**0.000**	**− 0.42**	**− 0.23**	− 0.07	− 1.63	0.103	− 0.17	0.02	0.00	− 0.12	0.908	− 0.09	0.08	
Ears	− 0.05	− 0.91	0.362	− 0.16	0.06	**− 0.25**	**− 4.85**	**0.000**	**− 0.35**	**− 0.15**	− 0.06	− 1.29	0.197	− 0.15	0.03	
Hands	0.04	0.84	0.404	− 0.06	0.15	0.07	1.39	0.166	− 0.03	0.17	**− 0.27**	**− 6.20**	**0.000**	**− 0.36**	**− 0.19**	
Legs	− 0.13	− 2.82	0.005	− 0.22	− 0.04	− 0.06	− 1.29	0.196	− 0.14	0.03	− 0.03	− 0.85	0.394	− 0.11	0.04	
Nose	0.04	0.89	0.376	− 0.05	0.14	− 0.04	− 0.80	0.424	− 0.13	0.06	− 0.04	− 1.05	0.295	− 0.13	0.04	
Mouth	− 0.06	− 1.12	0.262	− 0.17	0.05	− 0.11	− 2.26	0.024	− 0.21	− 0.02	− 0.04	− 0.78	0.436	− 0.12	0.05	
Eyes * age	0.00	0.43	0.668	0.00	0.01	0.00	0.64	0.520	0.00	0.01	0.00	1.75	0.081	0.00	0.01	
Ears * age	0.00	0.15	0.883	− 0.01	0.01	0.00	0.26	0.791	− 0.01	0.01	0.00	− 0.28	0.777	− 0.01	0.00	
Hands * age	0.00	0.32	0.752	− 0.01	0.01	0.00	0.19	0.853	− 0.01	0.01	0.00	1.67	0.096	0.00	0.01	
Legs * age	0.00	0.36	0.720	0.00	0.01	0.00	− 1.64	0.101	− 0.01	0.00	0.00	− 0.67	0.506	− 0.01	0.00	
Nose * age	0.00	− 0.95	0.342	− 0.01	0.00	0.00	0.15	0.880	0.00	0.01	0.00	− 1.77	0.078	− 0.01	0.00	
Mouth * age	0.00	− 0.77	0.440	− 0.01	0.00	0.00	0.27	0.789	− 0.01	0.01	0.00	− 1.32	0.187	− 0.01	0.00	

Target: agency	Legs					Nose					Mouth					
Model	R2	F-value	p-value			R2	F-value	p-value			R2	F-value	p-value			
	0.30	21.49	0.000			0.19	12.45	0.000			0.15	9.65	0.000			
Explanatory variables: discomfort	β	t-value	p-value	95% CI		β	t-value	p-value	95% CI		β	t-value	p-value	95% CI		VIF
Eyes	0.01	0.18	0.859	− 0.07	0.09	− 0.06	− 1.29	0.199	− 0.16	0.03	− 0.02	− 0.39	0.700	− 0.11	0.07	2.07
Ears	− 0.08	− 1.63	0.104	− 0.17	0.02	− 0.06	− 1.05	0.294	− 0.17	0.05	− 0.09	− 1.66	0.097	− 0.19	0.02	2.10
Hands	0.04	0.91	0.362	− 0.05	0.13	0.07	1.33	0.183	− 0.03	0.18	0.04	0.75	0.451	− 0.06	0.13	2.53
Legs	**− 0.39**	**− 10.09**	**0.000**	**− 0.47**	**− 0.31**	− 0.10	− 2.08	0.038	− 0.19	− 0.01	− 0.07	− 1.61	0.109	− 0.15	0.02	2.13
Nose	− 0.04	− 0.93	0.354	− 0.12	0.04	**− 0.35**	**− 6.84**	**0.000**	**− 0.45**	**− 0.25**	− 0.07	− 1.47	0.143	− 0.16	0.02	1.88
Mouth	− 0.02	− 0.52	0.604	− 0.11	0.07	− 0.01	− 0.13	0.898	− 0.11	0.10	**− 0.23**	**− 4.60**	**0.000**	**− 0.33**	**− 0.13**	2.04
Eyes * age	0.00	0.61	0.540	0.00	0.01	0.00	1.42	0.155	0.00	0.01	0.00	0.61	0.539	0.00	0.01	1.91
Ears * age	0.00	0.70	0.482	0.00	0.01	0.00	0.14	0.890	− 0.01	0.01	0.00	0.21	0.830	− 0.01	0.01	2.16
Hands * age	0.00	0.96	0.337	0.00	0.01	0.00	0.21	0.832	− 0.01	0.01	0.00	0.57	0.567	0.00	0.01	2.60
Legs * age	0.00	− 0.32	0.751	− 0.01	0.00	0.00	− 0.60	0.550	− 0.01	0.00	0.00	− 0.47	0.639	− 0.01	0.00	2.13
Nose * age	0.00	− 1.17	0.242	− 0.01	0.00	0.00	− 1.39	0.165	− 0.01	0.00	0.00	− 1.02	0.310	− 0.01	0.00	1.81
Mouth * age	0.00	− 1.14	0.254	− 0.01	0.00	0.00	− 0.13	0.899	− 0.01	0.01	0.00	− 0.23	0.819	− 0.01	0.01	2.38

Bold letters indicate significant beta values.

#### Internal configurations

**Figure 4:**
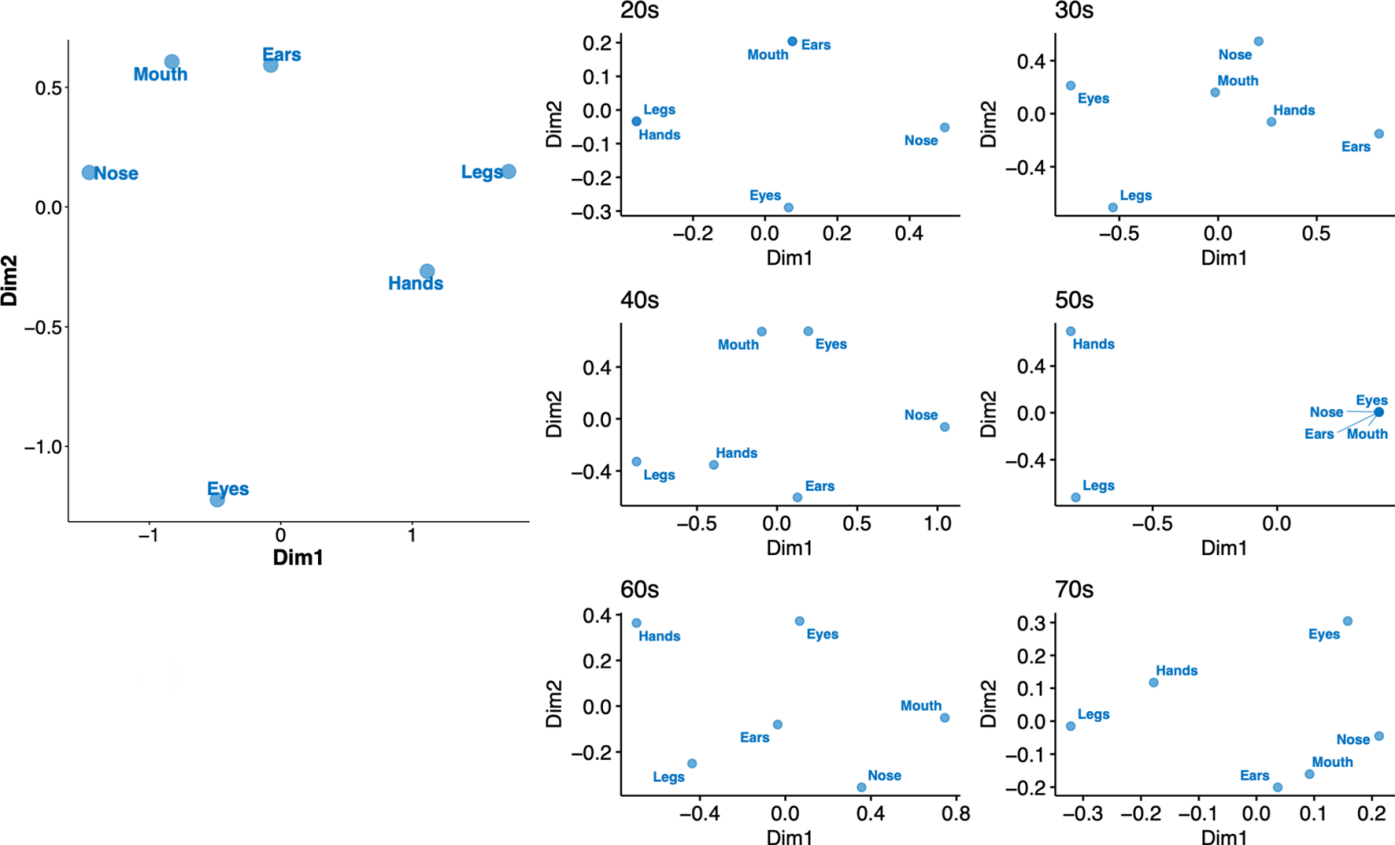
Internal configurations of the body parts with multidimensional scaling analyses for discomfort. The left-side panel shows the whole participants’ data and right-side panels show each age group data. Plots were generated using R software version 4.0.1 (R Core Team (2020). R: A language and environment for statistical computing. R Foundation for Statistical Computing, Vienna, Austria. http://www.R-project.org/).

We performed the MDS analyses by evaluating the relative distance of each target pair. All participants’ data showed that the body parts were sparse in a 2-D space (Fig. 4). The same tendency was observed for each age group data except for the 50s age group data. For 50s age group data, the hands and legs were each separated from the other body parts.

### IADL and orthotic equipment

The distribution of IADL scores showed that almost all participants’ scores (90.8%) were the maximum (13) (Supplementary Figure S7, left). The majority of our participants thus did not explicitly report any problems regarding daily activities. As for the use of orthotic equipment, our participants reported the use of eye equipment (eyeglasses, contact lens, and reading glasses), ear equipment (hearing aid, only two data), and others (orthodontics and crutch, only two data). The use of eye equipment was equally reported among all age groups (Supplementary Figure S7, right).

## Discussions

This survey study exploratory investigated the characteristics of bodily ownership and agency sensations of multiple body parts without experimental manipulations for a large population-based sample (*N* = 580). Our results showed that there exist some unique characteristics for bodily sensations in a natural state.

### Relationships regarding the bodily ownership and agency sensations

Ownership and agency sensations were positively related to each other in each body part (Tables 1 and 2). Experimental studies of the body transfer illusions in a single body part have demonstrated that the ownership and agency sensations positively interacted when the participants’ active body movements were synchronized with dynamic visual feedback on a fake body part^22,23,25,26^. Our findings suggest that the bodily ownership and agency sensations of each body part are positively associated with each other in a natural, daily life state where our body movements and visual feedback from our body sites are always congruent.

Notably, the current study further demonstrated that the agency sensations of the hands and legs were positively related to the ownership sensations of all body parts (Table 2). Stronger agency sensation was reported to enhance ownership sensation during RHI^22,29^. Furthermore, illusory body ownership was shown to occur for an invisible body by visual-motor synchrony between the movements of virtual hand/feet and those of the participant’s body in a virtual reality situation^17^. One may consider that, in addition to the hands and legs, our eyes and mouths also move in daily life. However, the agency sensations of the hands and legs may reach our consciousness more frequently simply because the movements of these body parts are also explicitly visible, whereas those of the eyes and mouth are often done unconsciously^e.g.,^^59^. We speculate that the agency sensations of the hands and legs, whose movements we may be more clearly conscious of, generally contribute to feeling ownership sensation in our whole body during daily life due to visual-motor synchrony.

The current study showed that the magnitude of ownership and agency were generally high for all targets. In our data, 102 out of 580 participants marked “strongly agree” ratings for all ownership and agency items on all targets. Given that the close and mutual relationships between the ownership and agency sensations^8,10,22^, these responses are assumed to be plausible. One may wonder why the agency scores were high even for typically immobile body parts, such as the ears and nose. However, it is reasonable to feel agency sensations for these body parts simply because we usually feel active, control sensations for these sensory organs, such as “listening” and “sniffing.” Our data also demonstrated that high ownership and agency sensations were reported for daily-use goods (shoes and clothes). Further, we found that the magnitudes of ownership and agency for the daily-use goods were greater for elder participants. Neurophysiological and behavioral studies have demonstrated that using tools can induce plastic changes in perceptual and neural processing, such that tools are represented as a part of our body^60^. Based on this, it is reasonable that we may feel high ownership and agency sensations for daily-use goods and that elder people can feel stronger ownership and agency sensations for daily-use goods with a longer duration of use. However, we should note that the effects of age were not observed for the ownership and agency of body parts. This indicates that ownership and agency sensations may qualitatively differ between body parts and external objects. These possibilities should be tested in future studies.

The current study measured the magnitude of discomfort (pain, numbness, or tremors) in each body part. The discomfort scores were negatively related to the ownership and agency sensation in each body part (Tables 3 and 4). It has been reported that lesser illusory bodily ownership sensation occurred when a virtual body had a uncomfortable body posture^61^ and that the degradation of agency sensation by hand immobilization induced a stronger RHI for that hand^39^. Moreover, a stronger RHI was reported to be positively associated with a lesser pain sensation^62^ ^but see also^ ^63^. We can consider that feeling discomfort in a body part weakens the bodily ownership and agency sensations on that body part in a natural, daily life state. We further found that ear discomfort was negatively associated with the ownership of eyes and hands. In our perception, sounds usually interact with visual information and touch^64,65^. Specifically, sounds are perceived as a consequence of visual (speaking voice) or tactile events (pushing buttons). Our findings may reflect the relationship between discomfort and bodily ownership in such daily life situations. We should note that we were unable to conclude whether the relationships between discomfort and bodily ownership and agency sensations are direct or not with our current data. The current study presented the discomfort sensation as pain, numbness, or tremors to the participants. It is possible that the discomfort sensations reflected dysfunctions of a body part, such as restrictions of control or movements, and these dysfunctions would lower ownership and agency sensations. Future studies should artificially introduce pain or numbness and investigate whether and how discomfort sensations can solely and directly modulate bodily ownership and agency sensations.

It has been reported that the different brain areas independently contribute to each bodily ownership and agency sensation^24^. Premotor cortex, intraparietal sulcus, and extrastriate body area are reported to be responsible for bodily ownership sensations, whereas the sensorimotor areas and parietal cortex around midline cortical structures are related to the agency sensation^8,24,66,67^. Our current findings would suggest that there exists each body part’s specific functional connectivity between the ownership- and agency-responsible brain regions. Further, the subregions related to the agency sensation of the hands and legs may have neural connections to each brain area responsible for the ownership sensation in each body part. We can also predict that the cortical and subcortical brain regions are related to somatosensory discomfort such as pain^68^ and may have inhibitory neural modulations in the ownership- and agency-responsible brain regions. Future neuroimaging studies can examine these possibilities.

### Effects of demographic variables

We did not find any significant relationships between the ownership and agency sensations of the body parts and participants’ gender or age. The impressions of the body such as attractiveness and appearance are reported to be different across gender and age^32,33^. Bodily ownership and agency sensations could be distinguished from these aesthetic evaluations. Some experimental studies reported incongruent findings regarding the effects of gender^6,29^ and age^19–21,34–37^ on illusory bodily transfer sensations. Our current survey study found no effects of age and gender on bodily ownership and agency sensations. We can speculate that the inconsistencies in the previous experimental findings may be due to individual differences in susceptibility to experimental manipulations. As discussed above, the discomfort sensations might also modulate the susceptibility of bodily ownership and agency sensations.

We observed some effects of the participant’s age on the discomfort sensation. Regarding the relationships between the discomfort sensation and age (Supplementary Table S13), nose discomfort showed a stronger effect on nose ownership for elderly participants. Further, we found that discomfort in the hands had a stronger effect on nose ownership for younger participants. We also observed that the discomfort score for eyes had a stronger effect on mouth ownership for younger participants. We found that the daily activity level was preserved and comparable among the participants based on the IADL scores. Although our data suggest that the participant’s age may have modulatory effects on the relationship between discomfort and ownership sensations, especially for some facial parts, our data cannot identify what causes these relationships. Further studies are thus required to understand these relationships, including detailed investigations of the nature and origin of discomfort sensations.

### Internal configurations of bodily sensations and discomfort

We found age-related differences regarding the internal configurations of the multiple body parts for the ownership and agency sensations estimated by MDS. The internal configurations for the ownership sensation (Fig. 2A) revealed that the ownership sensations were grouped among the body parts and separated from those of the goods for all participants’ data and almost all age group data. However, the ownership sensations of the body parts were sparsely distributed for the 60s age group data. Similarly, regarding the agency sensation (Fig. 2B), we found that some body parts and goods are located together in one dimension in the 60s age group, but the other data showed a clear separation between the body parts and goods. These results suggest that the changes in the internal configurations of the multiple body parts for the ownership and agency sensations occur in the 60s age group. It may be notable that the alteration of the internal configurations for discomfort was observed in the 50s age group (Fig. 4): the hands, legs, and other body parts were each separately located, whereas the body parts were sparse in the maps for the other data. We may assume that discomfort sensation due to muscle attenuation, dysfunction of body joints, and pain frequently appear in one’s 50s in the hands, legs, and other body parts independently. These would induce alterations in the internal configurations of the ownership and agency sensations across the body parts. These possibilities can be tested by, for example, a longitudinal questionnaire study.

### Summary

The current study demonstrated that bodily ownership and sensations are measurable, and the characteristics of these sensations can be investigated using the survey method without any experimental manipulations. Our results demonstrated the positive relationships between the ownership and agency sensations in each body part. We further found that the stronger agency sensations of the hands and legs contributed to the higher ownership sensations of the other body parts. In contrast, the discomfort sensation was negatively associated with each bodily sensation. We also found the unique aspects of the qualitative internal configurations of the bodily sensations of an age group (60s).

Whereas the current study simply asked our participants to complete the online survey, the manner in which participants completed their responses may have affected their online bodily sensations. For example, we may need to consider whether they used a mouse with a PC or their fingers with a smartphone/tablet and whether they were sitting or standing/walking. Future studies should control and evaluate these aspects as covariates. By considering the evidence of body transfer illusions on the whole body^13–17^ and close relationships between interoception and self-consciousness^69^, future studies may need to include central body segments, such as the trunk and back, and internal organs, such as the heart, as targets. The current study defined “hands” as the region from the shoulder to the fingertips and “legs” as the region from the hip to the toes. Since our results demonstrated strong contributions from the agency sensations of these body parts on the ownership sensations of all body parts, the role of each segment of the hands and legs should be investigated in detail in the future. Since the findings in the current study are limited to Japanese samples, performing a cross-cultural study can also be useful to test the replicability and validity as well as the culture-dependency of the bodily sensations.

## Supplementary Information

Supplementary Information.

## Data Availability

Our data and analysis code have been made publicly available via the Open Science Framework and can be accessed at https://osf.io/6bxpe/.
