# Supplementary material for: Bodily ownership and agency sensations in a natural state
Source: Sci Rep. 2021 Apr 21;11:8651. doi: 10.1038/s41598-021-87843-2 (PMC8060257; doi:10.1038/s41598-021-87843-2)
Supplement: Supplementary file 1 — Supplementary Information. [file 41598_2021_87843_MOESM1_ESM.pdf]

Supplementary Information

Bodily ownership and agency sensations in a natural state

Souta Hidaka[1 #], Kyoshiro Sasaki[2], Toshikazu Kawagoe[1], Nobuko Asai[3], and Wataru Teramoto[4]

1. Department of Psychology, Rikkyo University, 1-2-26, Kitano, Niiza-shi, Saitama, 352-8558 Japan.

2. Faculty of Informatics, Kansai University, 2-1-1, Ryozenji-cho, Takatsuki, Osaka, 569-1095, Japan.

3. Faculty of Social Relations, Kyoto-Bunkyo University, 80 Senzoku, Makishima-cho, Uji-city, Kyoto, 611-00414.

4. Department of Psychology, Kumamoto University, 2-40-1 Kurokami, Chuo-ku, Kumamoto 860-8555

*Corresponding author:

Souta Hidaka

E-mail: hidaka@rikkyo.ac.jp

Address: Department of Psychology, Rikkyo University, 1-2-26, Kitano, Niiza-shi, Saitama, 352-8558 Japan.

Supplementary information S1. English-Japanese questionnaire items for ownership, agency, and discomfort.

English	Japanese
**Ownership**	
I feel like it is my own	私は、それを自分自身のものであると感じる
I feel like it is not mine	私は、それを自分自身のものであると感じない
I feel like it is somebody else's	私は、それを他人のものであるように感じる
**Agency**	
I feel like I am in control of it	私は、それを思い通りに使うことが出来ると感じる
I feel like it is out of my control	私は、それを思い通りに使うことが出来ないと感じる
**Discomfort**	
How much discomfort (such as pain, numbness, or tremors) do you feel?	私は、それに違和感(例えば痛み、しびれ、ふるえなど)を感じる

Ownership to Agency

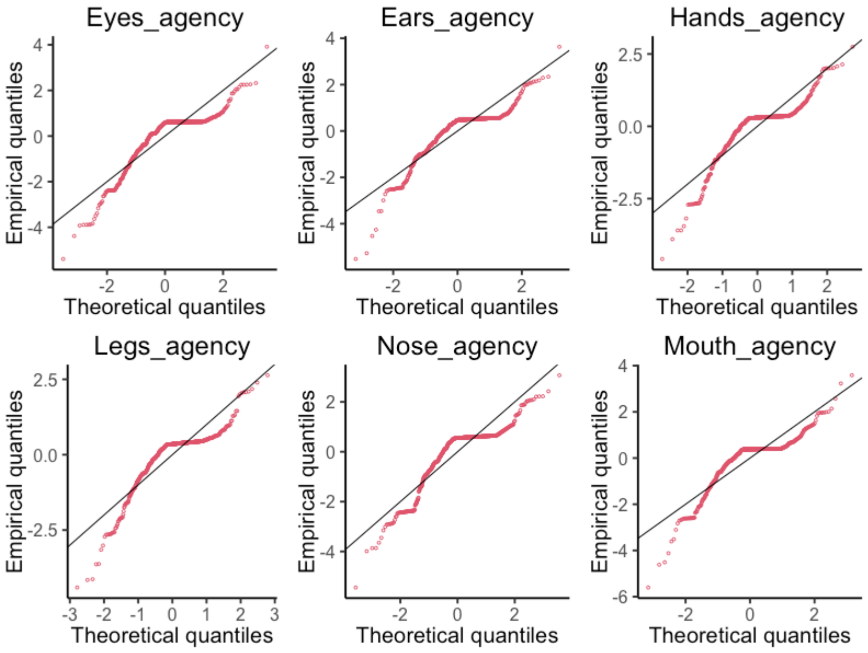

Agency to ownership

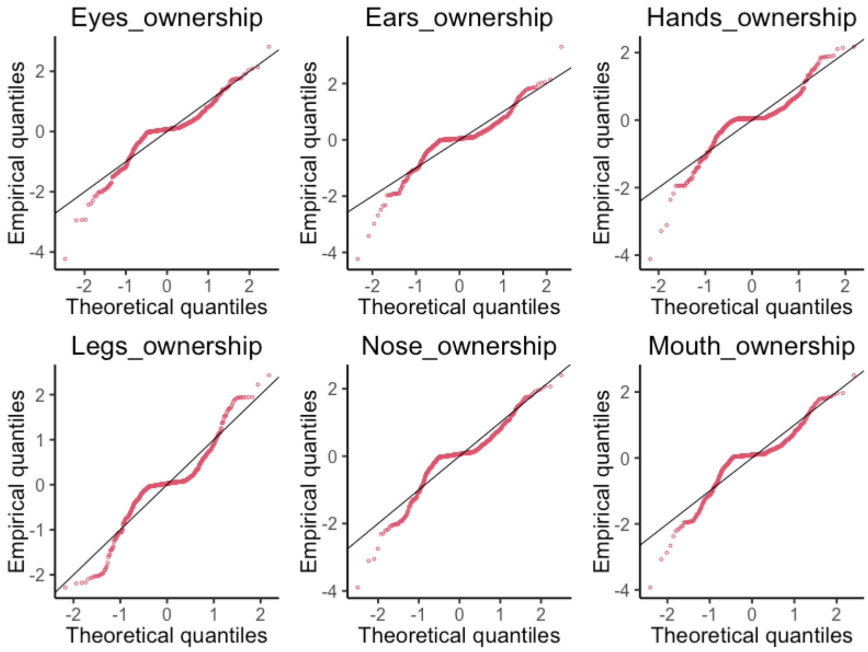

Supplementary figure S1. Q-Q plots for multiple regression analyses between the ownership and agency sensations. Plots were generated using R software version 4.0.1 (R Core Team (2020). R: A language and environment for statistical computing. R Foundation for Statistical Computing, Vienna, Austria. http://www.R-project.org/).

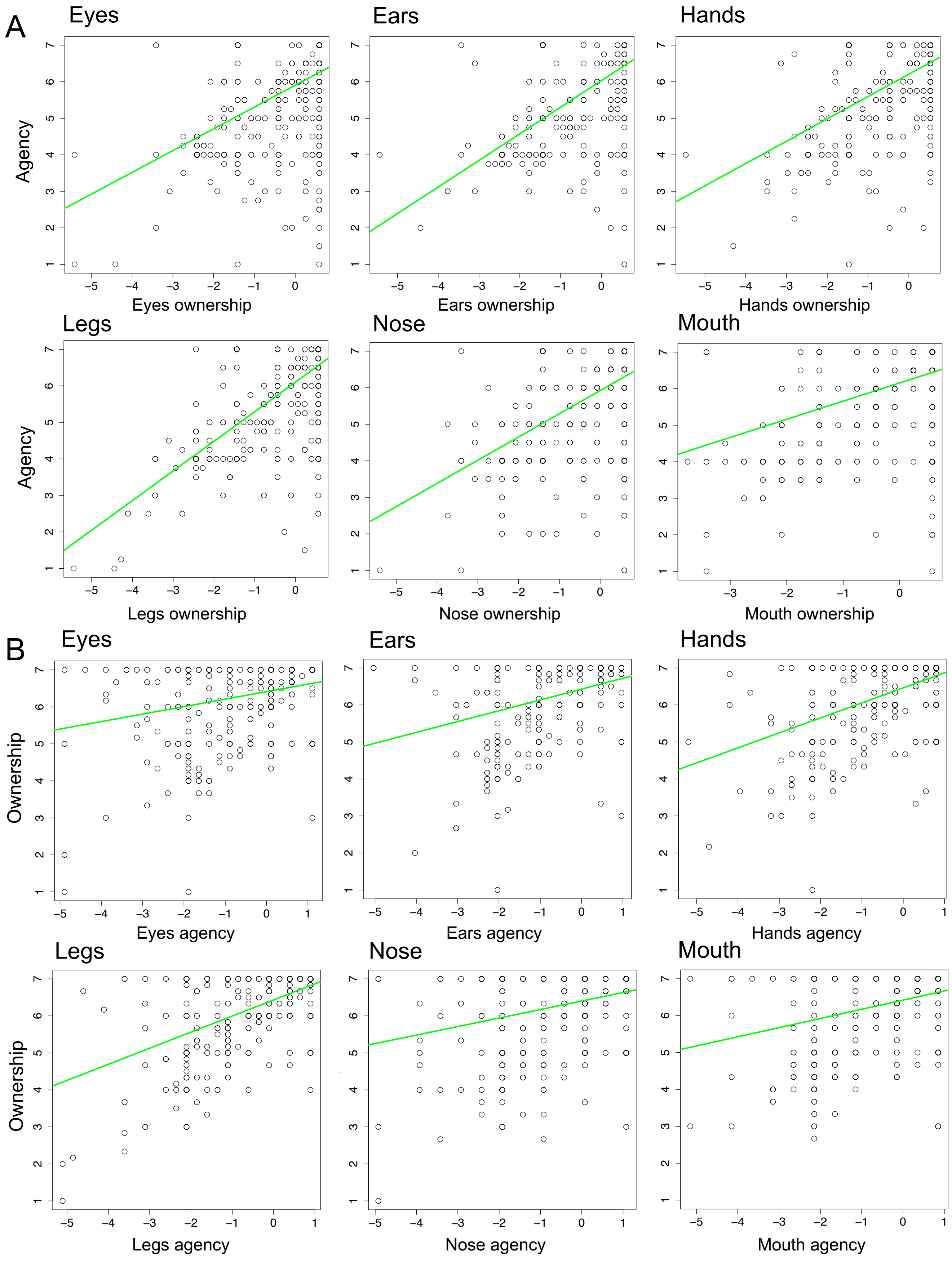

Supplementary figure S2. Scatter plots with regression lines (green) for the multiple regression analyses from (A) the ownership to the agency sensations and (B) the agency to the ownership sensations in each body part. Plots were generated using R software version 4.0.1 (R Core Team (2020). R: A language and environment for statistical computing. R Foundation for Statistical Computing, Vienna, Austria. http://www.R-project.org/).

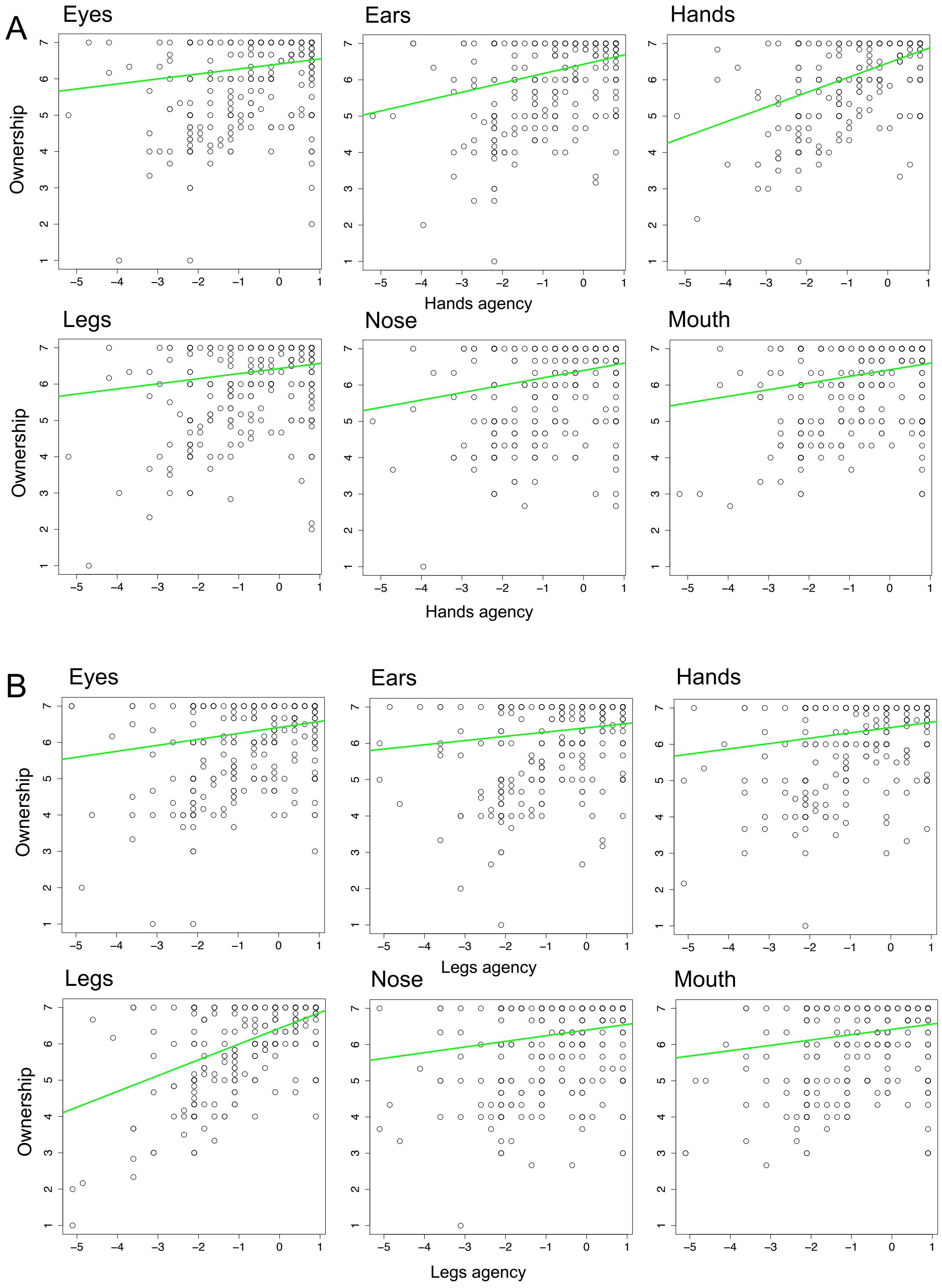

Supplementary figure S3. Scatter plots with regression lines (green) for the multiple regression analyses (A) from the agency of hands and (B) from the agency of legs to the body parts. Plots were generated using R software version 4.0.1 (R Core Team (2020). R: A language and environment for statistical computing. R Foundation for Statistical Computing, Vienna, Austria. http://www.R-project.org/).

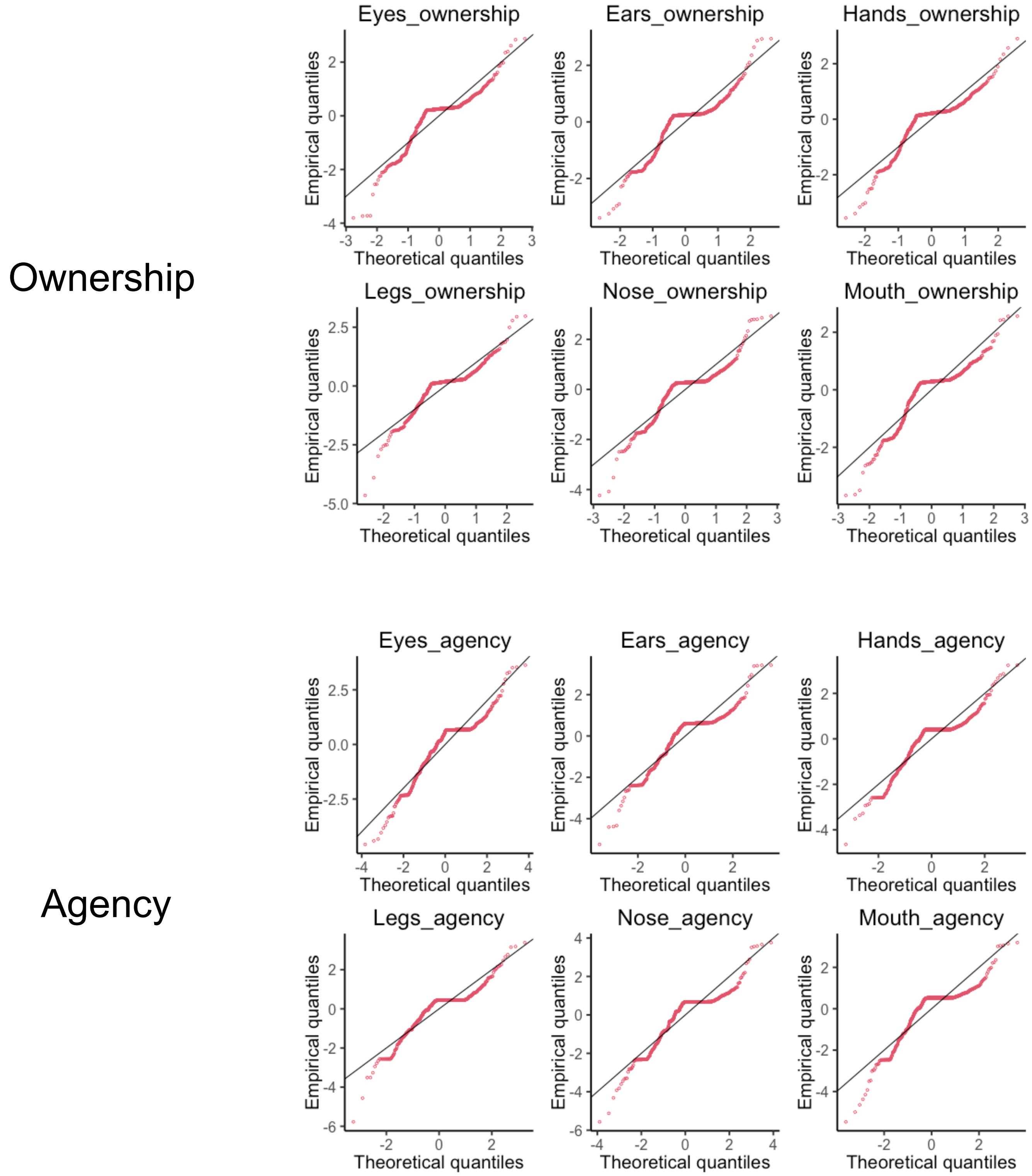

Supplementary figure S4. Q-Q plots for multiple regression analyses from the discomfort to ownership sensation or agency sensation. Plots were generated using R software version 4.0.1 (R Core Team (2020). R: A language and environment for statistical computing. R Foundation for Statistical Computing, Vienna, Austria. http://www.R-project.org/).

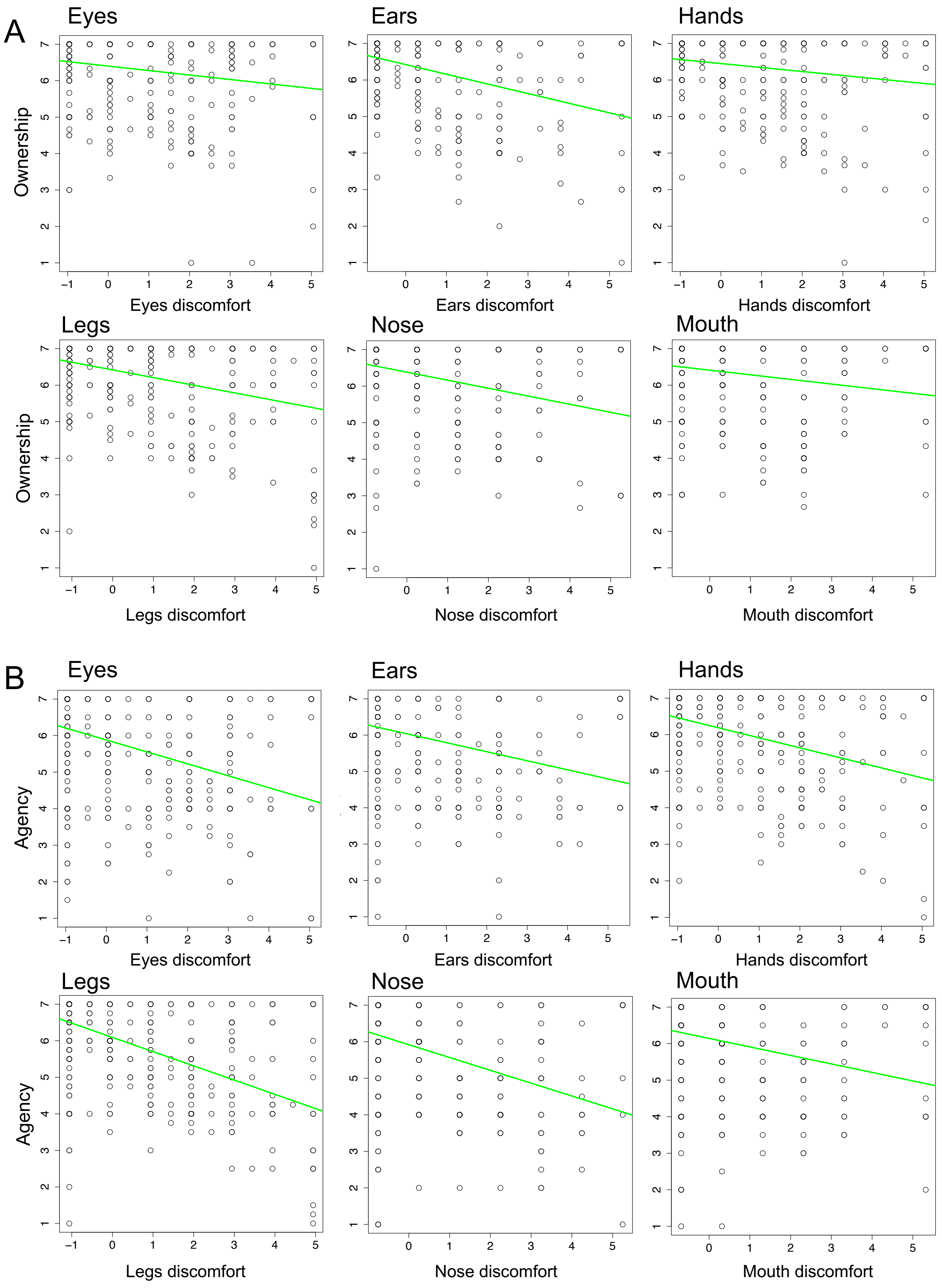

Supplementary figure S5. Scatter plots with regression lines (green) for the multiple regression analyses from discomfort to (A) the ownership and (B) agency sensations. Plots were generated using R software version 4.0.1 (R Core Team (2020). R: A language and environment for statistical computing. R Foundation for Statistical Computing, Vienna, Austria. http://www.R-project.org/).

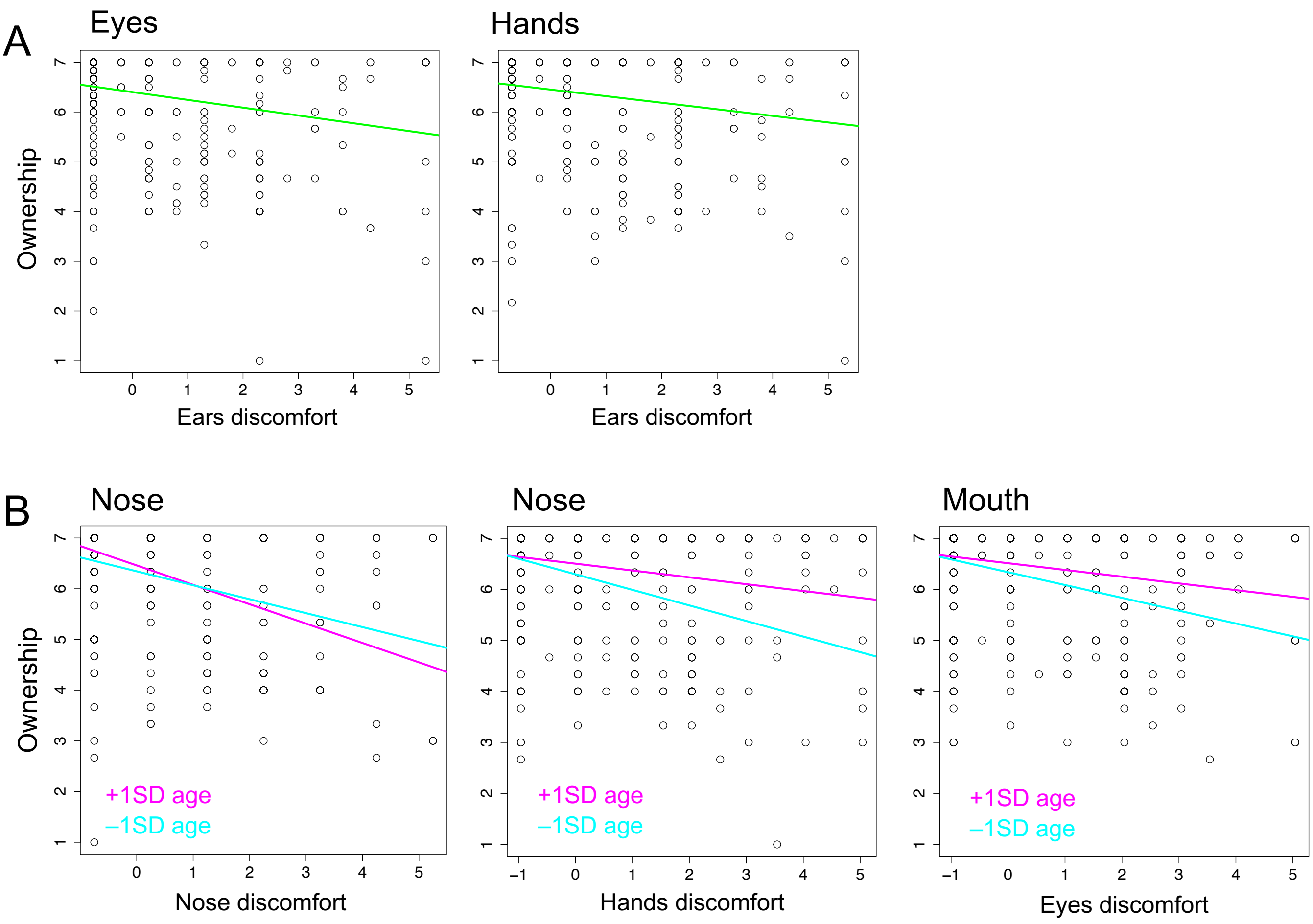

Supplementary figure S6. Scatter plots with regression lines (green) for the multiple regression analyses (A) from ears' discomfort to the eyes' and hands' ownership and (B) the interactions between the nose's discomfort or hands' discomfort to the nose's ownership and the eyes' discomfort to the mouth's ownership. Plots were generated using R software version 4.0.1 (R Core Team (2020). R: A language and environment for statistical computing. R Foundation for Statistical Computing, Vienna, Austria. http://www.R-project.org/).

A

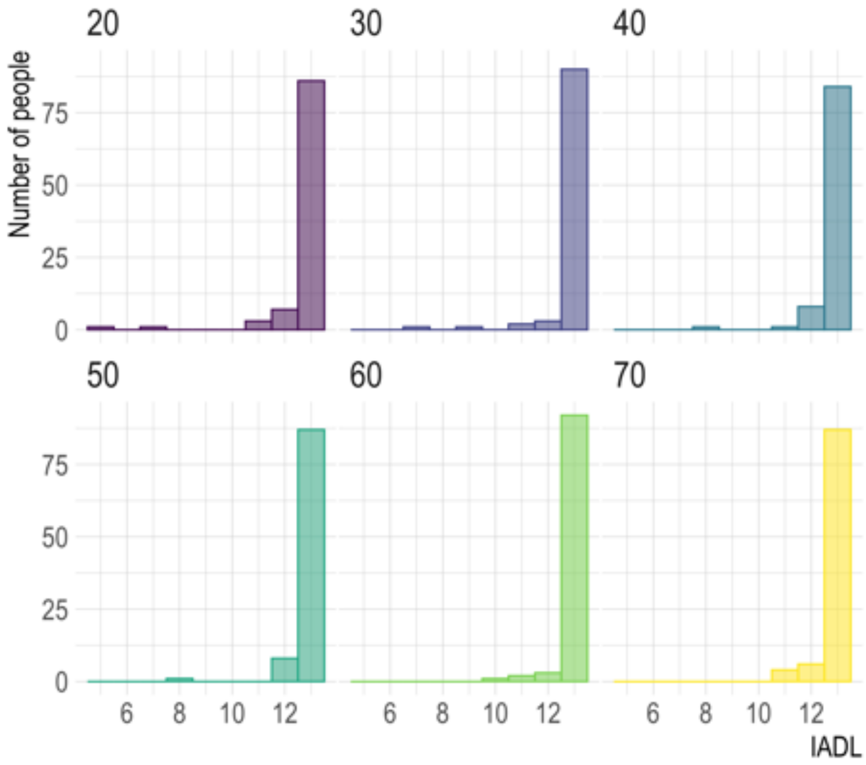

B

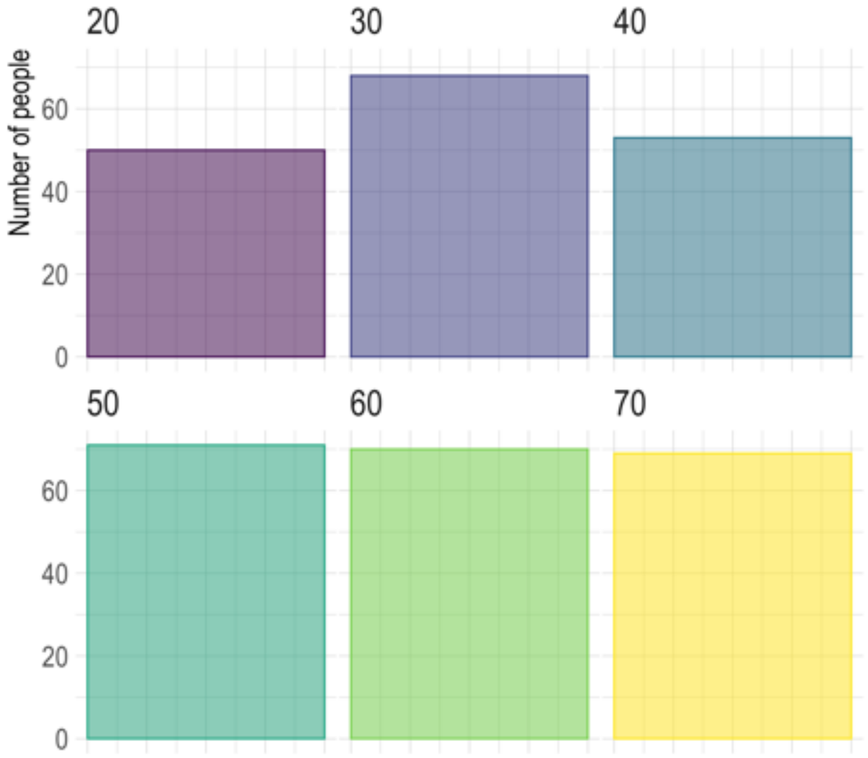

Supplementary figure S7. Histograms of (A) the IADL scores and (B) the use of eye equipment in each age group. Plots were generated using R software version 4.0.1 (R Core Team (2020). R: A language and environment for statistical computing. R Foundation for Statistical Computing, Vienna, Austria. http://www.R-project.org/).

Supplementary table S1. Statistics for the comparison of the laterality in each body part for the ownership and agency sensations.

	Ownership			Agency		
Target	W	pval	PS	W	pval	PS
Eyes	1006.50	0.406	0.05	1971.00	0.756	0.08
Ears	1074.00	0.650	0.06	1771.50	0.064	0.08
Hands	1004.00	0.978	0.06	2692.00	0.216	0.08
Legs	1145.00	0.707	0.06	2406.00	0.637	0.08

Supplementary table S2. Statistics for the normality tests in each body part for the ownership and agency sensations.

	Ownership		Agency	
Target	W	pval	W	pval
Eyes	0.65	0.000	0.79	0.000
Ears	0.63	0.000	0.75	0.000
Hands	0.62	0.000	0.71	0.000
Legs	0.63	0.000	0.74	0.000
Nose	0.64	0.000	0.76	0.000
Mouth	0.64	0.000	0.71	0.000
Clothes	0.81	0.000	0.84	0.000
Shoes	0.80	0.000	0.82	0.000

Supplementary table S3. Statistics for the comparison of the gender in each body part for the ownership and agency sensations.

	Ownership			Agency		
Target	W	pval	PS	W	pval	PS
Eyes	39459.00	0.160	0.26	39374.00	0.184	0.34
Ears	39776.00	0.213	0.25	39874.00	0.279	0.32
Hands	39540.50	0.161	0.24	39178.50	0.136	0.30
Legs	40888.00	0.559	0.27	40874.50	0.586	0.33
Nose	40499.00	0.410	0.25	40176.00	0.358	0.32
Mouth	40696.50	0.483	0.26	41032.00	0.636	0.31
Clothes	41277.50	0.752	0.38	40798.50	0.575	0.38
Shoes	42211.00	0.864	0.38	41863.50	0.991	0.38

Supplementary table S4. Correlations between the years of education and the ownership sensation or agency sensation.

	Ownership		Agency	
Target	tau	pval	tau	pval
Eyes	0.03	0.423	-0.03	0.346
Ears	0.06	0.069	0.01	0.812
Hands	0.08	0.025	0.03	0.469
Legs	0.06	0.123	-0.01	0.676
Nose	0.05	0.194	-0.01	0.785
Mouth	0.06	0.107	0.01	0.810
Clothes	0.03	0.321	0.01	0.854
Shoes	0.05	0.161	0.02	0.510

Supplementary table S5. (Left) Correlations between age and the ownership sensation or agency sensation. (Right) Partial correlations between age and the ownership sensation or agency sensation including the years of education as a covariate.

Correlation	Ownership		Agency	
Target	tau	pval	tau	pval
Eyes	0.05	0.052	0.04	0.239
Ears	0.05	0.104	0.02	0.471
Hands	0.07	0.011	0.01	0.757
Legs	0.06	0.048	0.01	0.792
Nose	0.07	0.013	0.06	0.044
Mouth	0.07	0.007	0.07	0.018
Clothes	0.11	0.000	0.13	0.000
Shoes	0.13	0.000	0.12	0.000

Partial correlation	Ownership		Agency	
Target	tau	pval	tau	pval
Eyes	0.05	0.052	0.03	0.241
Ears	0.05	0.104	0.02	0.397
Hands	0.07	0.011	0.01	0.646
Legs	0.06	0.048	0.01	0.816
Nose	0.07	0.013	0.06	0.025
Mouth	0.07	0.007	0.08	0.006
Clothes	0.11	0.000	0.14	0.000
Shoes	0.13	0.000	0.13	0.000

Supplementary table S6. Correlations within and between the ownership and agency sensations.

		Agency								Ownership							
		Eyes	Ears	Hands	Legs	Nose	Mouth	Clothes	Shoes	Eyes	Ears	Hands	Legs	Nose	Mouth	Clothes	Shoes
Agency	Eyes	1.00															
	Ears	0.63	1.00														
	Hands	0.60	0.59	1.00													
	Legs	0.60	0.60	0.67	1.00												
	Nose	0.57	0.65	0.56	0.58	1.00											
	Mouth	0.60	0.60	0.63	0.63	0.63	1.00										
	Clothes	0.48	0.48	0.44	0.44	0.51	0.48	1.00									
	Shoes	0.51	0.55	0.52	0.48	0.56	0.52	0.64	1.00								
Ownership	Eyes	0.57	0.49	0.51	0.51	0.44	0.48	0.37	0.44	1.00							
	Ears	0.47	0.60	0.56	0.53	0.46	0.52	0.39	0.46	0.72	1.00						
	Hands	0.45	0.48	0.63	0.52	0.43	0.51	0.37	0.44	0.71	0.77	1.00					
	Legs	0.46	0.51	0.56	0.64	0.47	0.53	0.36	0.44	0.71	0.76	0.76	1.00				
	Nose	0.44	0.49	0.51	0.52	0.55	0.51	0.40	0.45	0.69	0.74	0.70	0.72	1.00			
	Mouth	0.46	0.45	0.52	0.52	0.45	0.58	0.40	0.43	0.70	0.72	0.72	0.72	0.71	1.00		
	Clothes	0.42	0.42	0.43	0.42	0.43	0.43	0.59	0.53	0.48	0.50	0.50	0.49	0.50	0.50	1.00	
	Shoes	0.41	0.46	0.44	0.44	0.45	0.44	0.50	0.63	0.48	0.53	0.50	0.52	0.51	0.50	0.66	1.00

Supplementary table S7. Statistics for the comparison of the laterality in each body part for discomfort.

Discomfort			
Target	W	pval	PS
Eyes	1095.50	0.629	0.05
Ears	622.00	0.402	0.04
Hands	1573.50	0.106	0.06
Legs	1466.50	0.857	0.07

Supplementary table S8. Statistics for the normality tests in each body part for discomfort.

Discomfort		
Target	W	pval
Eyes	0.68	0.000
Ears	0.59	0.000
Hands	0.67	0.000
Legs	0.69	0.000
Nose	0.61	0.000
Mouth	0.57	0.000

Supplementary table S9. Correlations of the discomfort between the body parts.

	Eyes	Ears	Hands	Legs	Nose	Mouth
Eyes	1.00					
Ears	0.57	1.00				
Hands	0.58	0.58	1.00			
Legs	0.52	0.56	0.63	1.00		
Nose	0.58	0.59	0.53	0.47	1.00	
Mouth	0.57	0.61	0.54	0.54	0.60	1.00

Supplementary table S10. Statistics for the comparison of the gender in each body part for discomfort.

		Discomfort	
Target	W	pval	PS
Eyes	46176.50	0.014	0.35
Ears	45623.00	0.021	0.29
Hands	44875.50	0.085	0.33
Legs	43776.00	0.287	0.33
Nose	45030.50	0.054	0.28
Mouth	45617.00	0.019	0.28

Supplementary table S11. Correlations between the years of education and discomfort.

	Discomfort	
Target	tau	pval
Eyes	-0.01	0.817
Ears	-0.03	0.482
Hands	-0.06	0.070
Legs	-0.04	0.280
Nose	0.02	0.631
Mouth	0.03	0.457

Supplementary table S12. (Left) Correlations between age and discomfort. (Right) Partial correlations between age and discomfort including the years of education as a covariate.

Correlation	Discomfort	
Target	tau	pval
Eyes	0.00	0.984
Ears	0.05	0.115
Hands	0.06	0.077
Legs	0.05	0.083
Nose	-0.04	0.167
Mouth	-0.04	0.216

Partial correlation	Discomfort	
Target	tau	pval
Eyes	0.00	0.990
Ears	0.05	0.083
Hands	0.05	0.078
Legs	0.05	0.069
Nose	-0.04	0.122
Mouth	-0.04	0.179

Supplementary table S13. Statistics of multiple regression analyses with significant interactions between discomfort and age

Target:<br>Nose ownership	Model:<br>Nose*age	R2	F-value	p-value		
		0.20	47.28	0.000		
Explanatory variables: Discomfort	β	t-value	p-value	95% CI		VIF
Nose(+1SD age)	**-0.38**	**-9.31**	**0.000**	**-0.46**	**-0.30**	2.17
Nose(-1SD age)	**-0.27**	**-7.52**	**0.000**	**-0.35**	**-0.20**	1.72
age (+1SD or -1SD)	0.00	1.49	0.138	0.00	0.01	1.01
Nose*age (+1SD or -1SD)	0.00	-2.00	0.046	-0.01	0.00	1.71

Target:<br>Nose ownership	Model:<br>Hands*age	R2	F-value	p-value		
		0.13	28.63	0.000		
Explanatory variables: Discomfort	β	t-value	p-value	95% CI		VIF
Hands(+1SD age)	**-0.13**	**-3.64**	**0.000**	**-0.21**	**-0.06**	1.99
Hands(-1SD age)	**-0.31**	**-8.26**	**0.000**	**-0.38**	**-0.23**	2.00
age (+1SD or -1SD)	**0.01**	**2.57**	**0.011**	**0.00**	**0.01**	1.00
Hands*age (+1SD or -1SD)	**0.01**	**3.29**	**0.001**	**0.00**	**0.01**	2.00

Target:<br>Mouth ownership	Model:<br>Eyes*age	R2	F-value	p-value		
		0.10	23.54	0.000		
Explanatory variables: Discomfort	β	t-value	p-value	95% CI		VIF
Eyes(+1SD age)	**-0.13**	**-3.47**	**0.001**	**-0.21**	**-0.06**	2.16
Eyes(-1SD age)	**-0.25**	**-7.32**	**0.000**	**-0.32**	**-0.18**	1.76
age (+1SD or -1SD)	0.01	2.25	0.025	0.00	0.01	1.00
Eyes*age (+1SD or -1SD)	0.00	2.37	0.018	0.00	0.01	1.76

Target:<br>Legs ownership	Model:<br>Mouth*age	R2	F-value	p-value		
		0.18	41.94	0.000		
Explanatory variables: Discomfort	β	t-value	p-value	95% CI		VIF
Mouth(+1SD age)	**-0.30**	**-7.03**	**0.000**	**-0.38**	**-0.21**	2.30
Mouth(-1SD age)	**-0.31**	**-8.19**	**0.000**	**-0.39**	**-0.24**	1.90
age (+1SD or -1SD)	0.00	1.65	0.099	0.00	0.01	1.00
Mouth*age (+1SD or -1SD)	0.00	0.30	0.762	0.00	0.00	1.90

# Bold letters indicate significant beta values.
